# Biomaterials Based on Organic Polymers and Layered Double Hydroxides Nanocomposites: Drug Delivery and Tissue Engineering

**DOI:** 10.3390/pharmaceutics15020413

**Published:** 2023-01-26

**Authors:** Vera Regina Leopoldo Constantino, Mariana Pires Figueiredo, Vagner Roberto Magri, Denise Eulálio, Vanessa Roberta Rodrigues Cunha, Ana Clecia Santos Alcântara, Gustavo Frigi Perotti

**Affiliations:** 1 Departamento de Química Fundamental, Instituto de Química, Universidade de São Paulo, Av. Prof. Lineu Prestes 748, CEP 05513-970, São Paulo 05513-970, SP, Brazil; 2 Instituto Federal de Educação, Ciência e Tecnologia de Mato Grosso (IFMT), Linha J, s/n–Zona Rural, Juína 78320-000, MT, Brazil; 3 Departamento de Química, Universidade Federal do Maranhão, São Luís 65080-805, MA, Brazil; 4 Instituto de Ciências Exatas e Tecnologia, Universidade Federal do Amazonas, Rua Nossa Senhora do Rosário, 3863, Itacoatiara 69103-128, AM, Brazil

**Keywords:** nanocomposites, layered double hydroxides, hydrotalcite, anionic clays, layered materials, intercalation compounds, composite biomaterials, nano-based drug carrier, drug delivery system, tissue engineering

## Abstract

The development of biomaterials has a substantial role in pharmaceutical and medical strategies for the enhancement of life quality. This review work focused on versatile biomaterials based on nanocomposites comprising organic polymers and a class of layered inorganic nanoparticles, aiming for drug delivery (oral, transdermal, and ocular delivery) and tissue engineering (skin and bone therapies). Layered double hydroxides (LDHs) are 2D nanomaterials that can intercalate anionic bioactive species between the layers. The layers can hold metal cations that confer intrinsic biological activity to LDHs as well as biocompatibility. The intercalation of bioactive species between the layers allows the formation of drug delivery systems with elevated loading capacity and modified release profiles promoted by ion exchange and/or solubilization. The capacity of tissue integration, antigenicity, and stimulation of collagen formation, among other beneficial characteristics of LDH, have been observed by in vivo assays. The association between the properties of biocompatible polymers and LDH-drug nanohybrids produces multifunctional nanocomposites compatible with living matter. Such nanocomposites are stimuli-responsive, show appropriate mechanical properties, and can be prepared by creative methods that allow a fine-tuning of drug release. They are processed in the end form of films, beads, gels, monoliths etc., to reach orientated therapeutic applications. Several studies attest to the higher performance of polymer/LDH-drug nanocomposite compared to the LDH-drug hybrid or the free drug.

## 1. Introduction

The world population reached the milestone of eight billion people, which brings challenges to achieving the goals of sustainable development addressed in Agenda 21 of the United Nations [1]. One target of the agenda includes the protection and promotion of human well-being and health status. In this context, biomaterials science has contributed to the development of new and/or superior therapeutical technologies in collaboration with other fields of science, including materials science, chemistry, biology, physics, engineering, pharmacy, and medicine.

According to a consensus conference supported by the International Union of Societies for Biomaterials Science and Engineering held in Chengdu, China, in 2018, the biomaterial is “*a material designed to take a form that can direct, through interactions with living systems, the course of any therapeutic or diagnostic procedure*” [2]. Hence, biomaterials include not only long-term medical implants such as heart valves or artificial joints but also materials on the nanoscale used for drugs, vaccines, gene delivery, tissue engineering and imaging contrast [3]. A biomaterial must show biocompatibility, a property defined as “*the ability of a material to perform its desired functions with respect to a medical therapy, to induce an appropriate host response in a specific application and to interact with living systems without having any risk of injury, toxicity, or rejection by the immune system and undesirable or inappropriate local or systemic effects*” [2]. Emphasis should be placed on the fact that the biocompatibility of a material depends on the host response in a specific application or situation, which means that the classification of a material as biocompatible deserves the specification of the application evaluated.

**Figure 1:**
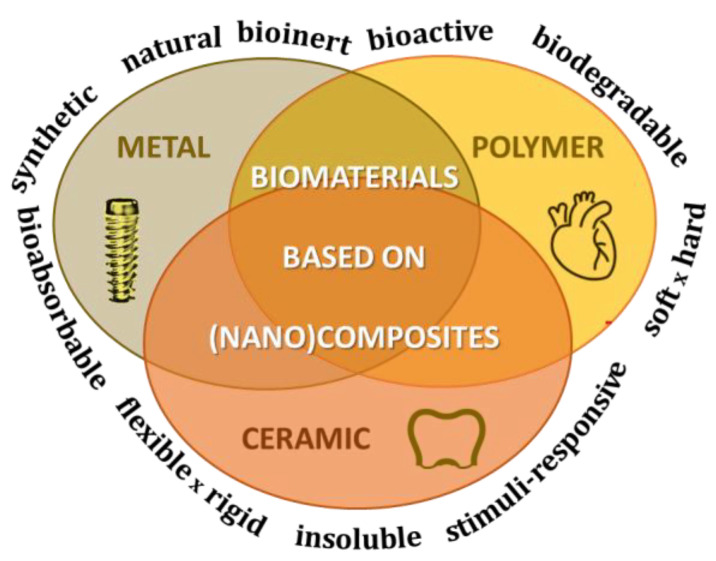
Classification of biomaterials by chemical composition. Materials having two or more phases are nominated (nano)composites. Biomaterials can have several properties guiding a particular application.

The application of biomaterials has highlighted their substantial role among pharmaceutical and medical strategies in the enhancement of quality of life. Considering the chemical composition, biomaterials are classified as metal, ceramic, polymer, or a mixture of them (Figure 1), i.e., a composite, defined by IUPAC (International Union of Pure and Applied Chemistry) as a “*multicomponent material comprising multiple, different (non-gaseous) phase domains in which at least one type of phase domain is a continuous phase*” [4]. Furthermore, if “*at least one of the phase domains has at least one dimension of the order of nanometers*”, the material is classified as a nanocomposite [4]. Biomaterial-based products are commercially available as, for instance, polymeric microspheres for hormone therapies [5].

Figure 1 indicates some properties of biomaterials, which diversity and complementarity allow combining phases of dissimilar chemical classes and length scales to obtain biomaterials showing improved or new physicochemical, mechanical and/or biological properties (i.e., synergic effects). Nature produces (nano)composites like bone and wood in a sophisticated hierarchical design [6], demonstrating concepts that inspire the scientific community and induce technological progress in diverse fields: biomedical, packing, textile, and aerospace, being fundamental to engender (nano)composites. In this way, biomaterials based on (nano)composites for pharmaceutical and biomedical applications should be tailored to match the properties required to reach the therapeutic goal. For instance, a pacemaker’s case of polymeric material is more suitable than the metal one because it is lighter and more resistant to corrosion [7]. For the rehabilitation of ligament injuries, this fibrous tissue can be repaired using a biodegradable composite of polylactic and hyaluronic acid esters. Issues related to sustainable processes of fabrication and social-economic cost-benefit also should be addressed when engendering biomaterials.

Over time, the properties required from a biomaterial have evolved from bioinerts (first generation), usual single-phase materials such as implants of titanium, to bioresponsives and bioresorbables ones (second generation) as bioglasses, to bioactive materials that provide biochemical stimuli (third generation), modulating the cell response (proliferation, migration, and differentiation) at the molecular level [8,9]. Considering the complexity of the desired properties, multifunctional (nano)composites are appropriate materials to accomplish such goals. They can combine the properties of the therapeutic agent as, for instance, antibacterial, antineoplastic, or antioxidant, promoting synergic effects. Furthermore, the materials should be responsive to intrinsic or external stimuli, triggering a specific biofunction. The material identifies a signal that can be a chemical, physical or biological/physiological stimulus such as, for instance, pH value, temperature, humidity, light, sound, electricity, magnetism, stress, reactive oxygen species (ROS), or enzyme activity [10,11]. Nowadays, materials with such properties are called "smart materials" or "smart devices", which include emerging materials (fourth generation) that recognize a particular disease in the beginning, revealing and treating it before any injury. The accomplishment of the fourth generation of biomaterials involves the concept of *biomaterialomics:* the materials’ design is based on the structure-property-functionality correlation extracted from experimental methods, advanced manufacturing, computational approach, database, and informatics tools [8]. 

**Figure 2:**
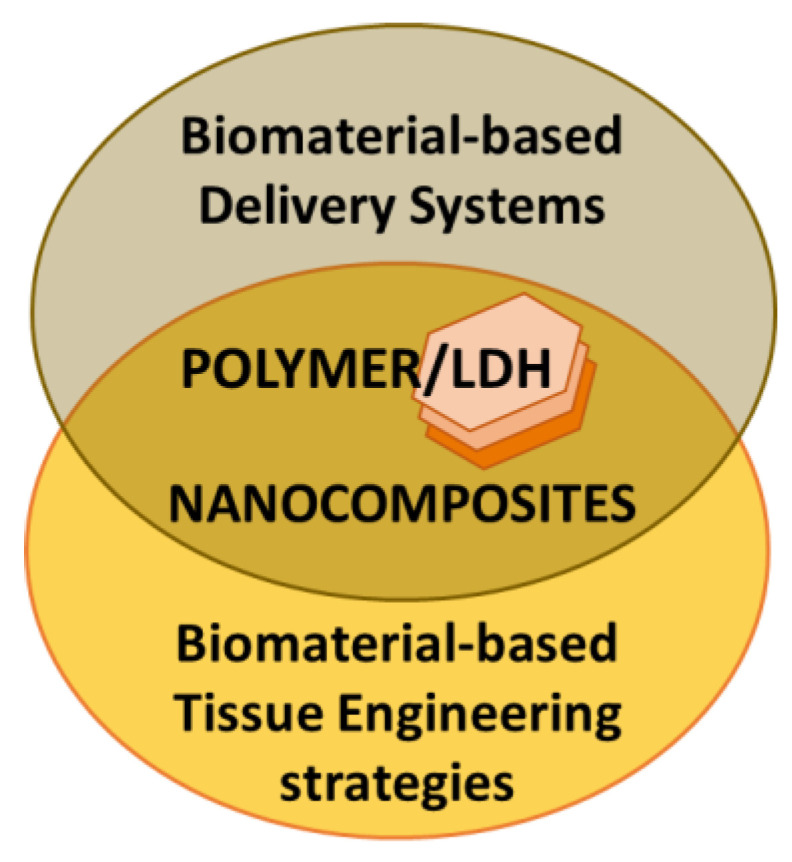
Biomaterials-based nanocomposites focused on this work: 2D nanomaterials and organic polymers.

This review focused on versatile biomaterials based on nanocomposites comprising a class of layered inorganic nanoparticles and natural or synthetic organic polymers aiming for drug delivery and tissue engineering purposes (Figure 2). Inorganic polymers, defined by IUPAC as “*polymer or polymer network with a skeletal structure that does not include carbon atoms*”, which comprise polyphosphazenes, polysilicates, polysiloxanes, polysilanes, polysilazanes, polygermanes, polysulfides etc. [4], are out of the scope of this review work.

The term tissue engineering is employed here in a comprehensive manner as a *discipline that seeks to repair, replace or regenerate tissues or organs by translating fundamental knowledge in physics, chemistry and biology into practical and effective materials or devices and clinical strategies* [12]. The inorganic material belongs to the class of layered double hydroxides (LDHs), whose structure and properties will be introduced ahead. Between the layers, LDHs can intercalate or confine simple anions (such as carbonate, nitrate, or chloride) or organic ions, metal complexes, and biopolymers, for instance. Since 2000, research works about the intercalation of drugs have addressed the development of drug delivery systems (DDS), as well as investigations to access the LDH biocompatibility [13]. 

In this work, the *drug* term was used in a broader meaning: a substance that has recognized therapeutic properties based on reported scientific investigations although not necessarily recognized in an official pharmacopeia for use as a medication. Moreover, the term *hybrid material* was employed as defined by IUPAC: “*material composed of an intimate mixture of inorganic components, organic components, or both types of component. The components usually interpenetrate on scales of less than 1 μm*” [4]. 

The studies about organic-inorganic hybrid materials in which the organic phase is a drug intercalated into the LDH inorganic phase (structural organization at the nanoscale) were not emphasized in this present review unless the hybrid material is dispersed in an organic polymeric phase. Several good review papers were published about drug-LDH nanohybrids [14,15,16,17,18,19,20,21,22,23,24,25].

## 2. Properties of Organic Polymers to Attend Pharmaceutical and Medicinal Applications

**Figure 3:**
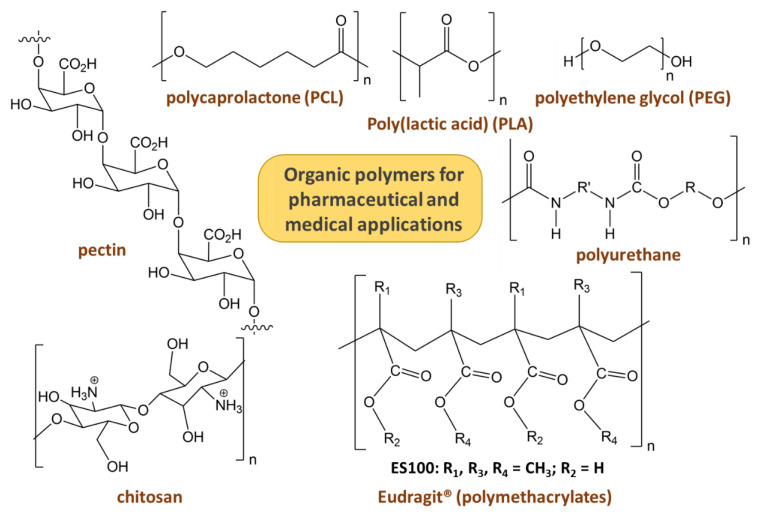
Chemical structures of some natural and synthetic organic polymers are used to develop biomaterial-based delivery systems and biomaterials for tissue engineering.

As shown in Figure 1, a biomaterial is classified as metal, ceramic, polymer, or a mixture of them. Some advantages of polymers are recognized compared to the other two types of materials: they can have several chemical compositions, different crystallinities and densities, and be molded with different shapes [26]. Consequently, their physical, chemical, mechanical and biological properties are more easily changeable according to the end application than metal or ceramics. Concerning the interest of polymers for pharmaceutical and medicinal issues, they have been applied in tissue engineering, DDSs, gene therapy, and bioimaging. Synthetic polymers obtained by controlled and reproductive processes and with a high level of purity to avoid unsafe effects seem to be more appealing than natural polymers. Both classes of polymers can be degradable or not, hard or soft, thermoplastic or thermosetting, swellable, hydrophilic or hydrophobic, among other properties, permitting a broad option of materials to line up with the expected application. For tissue engineering applications, biodegradable polymers are requested, which include polymers of natural origin such as the polysaccharides chitosan (CS) and pectin, biotechnological products such as poly(lactic acid) (PLA), and petrochemical-based polymers as polycaprolactone (PCL), polyethylene glycol (PEG) (see their structures in Figure 3) [27].

Polymers are also used as nanocarriers in DDSs: capsules having the drug dissolved or dispersed in the core or solid spheres. Usually, encapsulated species are controlled and released by diffusion, external stimuli, swelling, degradation, or chemical bond cleavage [28]. Besides the natural polymers CS and dextran, PLA and PCL, carriers from polyvinyl alcohol (PVA), PEG, polymethacrylates (commercial Eudragit class), and thermoplastic polyurethanes find application in DDS (structures shown in Figure 3).

Hydrophilic polymer (or co-polymer) can form a gel, defined as a *"non-fluid colloidal network or polymer network that is expanded throughout its whole volume by a fluid. A gel can contain a covalent polymer network, e.g., a network formed by crosslinking polymer chains or by non-linear polymerization; a polymer network formed through the physical aggregation of polymer chains caused by hydrogen bonds, crystallization, Helix formation, complexation, etc., that results in regions of local order acting as the network junction points"* [4]. Gels are molded in different aspects (micro- or nanoparticles, films, monoliths etc.), are stimuli-responsive (for instance, thermoreversible gel, which swollen is controlled by temperature variation), and can be nonionic, ionic, or amphoteric [29]. They present different swelling grades, being those with high levels (90–99.5%) are more biocompatible and preferred for medical applications. Hydrogels are appropriate to produce scaffolds for tissue engineering because they retain a high amount of water, have elevated porosity, and are easily molded in different shapes and sizes or directly injected into specific sites, i.e., they show ideal properties for cell growth and secretion of cellular matrix [30]. For bone formation, hydrogels should have a pore size in the range of 200–400 μm, while a pore size between about 90–200 μm is more appropriated for cartilage formation. The level of the hydrogel degradation (degradable, semi-permanent or permanent) depends on the application, type of injury etc. The biodegradable polymers used in hydrogels are homopolysaccharides (cellulose, starch etc.), heteropolysaccharides (CS etc.), peptides/proteins (gelatin, albumin, silk etc.), and deoxyribonucleic acid (DNA) and ribonucleic acid (RNA) [29]. Among the synthetic polymers, non-biodegradable (2-hydroxypropyl methacrylamide; poly(ethylene glycol) monoacrylate and biodegradable (PEG) polymers are used as hydrogels. The physicochemical properties of hydrogels can be enhanced if composed of natural and synthetic polymers because they align the advantages of each class of macromolecules (for instance, the hybrid hydrogel can respond in vivo to biological signals or remote triggers) [29]. Besides tissue engineering, hybrid hydrogels are applied for drug-controlled release, wound dressing, and ophthalmic lenses, among other uses.

Some characteristics of polymers used in pharmacy and medicine should be highlighted. Chitosan (and its structure-modified derivatives) has antimicrobial properties, immunostimulant effects, hemostatic function, antitumor and anti-inflammatory activities [31]. The polymer is obtained from crustaceous, and samples with low mass weight usually have the best bioactivity. Physical and mechanical properties of CS-based polymers permit their processing by several methods, manufacturing films, hydrogels, microparticles or nanofibers; CS-based health materials are available in the market. When applied to the production of hydrogel scaffolds for bone and cartilage regeneration, CS is associated with other polymers to reach the expected mechanical strength [30]. Equally CS, pectic polysaccharides have several similar pharmacological properties but show higher structural complexity [32]. They are obtained from plants (for instance, apple or lemon peel and sugar beet), which means effective accessibility. Pectin-based polymers can be used as DDS molded as beads, films, or gel capsules. Pectin forms gel in acidic media is dissolved in basic media, and can be chemically modified by reactions involving carboxylic and alcohol groups, for instance, promoting changes in its formal charge, degree of esterification, and molecular mass [33].

PEG is a synthetic, amphiphilic, and water-soluble polymer composed of ethylene glycol units [34]. It is a polymer of choice due to its low toxicity, biological inertness, and versatility. Plenty of PEG with different molecular mass and size chains, as well as PEG derivatives and co-polymers, are available for DDS and pharmaceutical purposes. It is noteworthy that the physicochemical properties and therapeutic efficiencies of PEG are highly dependent on its molecular weight. The surface modification of nanoparticles is achieved by PEGylation (grafting) through covalent or non-covalently linkage with PEG assuming different conformations and packing degrees according to the preparation method [34]. The polymer provides steric stability to nanoparticles, improving the pharmacokinetics and pharmacodynamics of drugs by avoiding or attenuating immunogenicity (escape the immune defense system) and antigenicity, combined with the improvement of the drug residence time and biodistribution. Formulations handled by oral, topical, and intravenous administration routes are reported, but the maximum dose to be administered is variable. A set of United States Food and Drug Administration (FDA)-approved therapeutic formulations constituted by PEG are available in the market to treat different diseases and illnesses [34,35], and it is also used as an excipient of SARS-CoV-2 Virus (COVID-19) vaccines [36]. Despite this, anti-PEG antibodies were noticed in the population that received the treatments, which can accelerate the drug/therapeutic clearance [34,35]. However, hypersensitivity and anaphylactic shock are some side effects that are rarely reported.

Commercial Eudragit^®^ polymers belong to the synthetic polymethacrylate class, and their properties depend on the substituent groups R in the carbon chains (Figure 3) [37]. They can be neutral, anionic, or cationic; the presence of carboxyl or amine groups allows the polymer solubilization in different pH ranges by protonation/deprotonation reactions, suitable for enteric coating applications. Some Eudragit^®^ samples are insoluble and exhibit pH-independent swelling properties; they are used for the development of sustained DDS materials. Thermoplastic medical-grade polyurethanes are used in commercial products: vaginal rings, stents, breast implants, heart valves, pacemaker leads, and also in medical thin-walled tubes and vascular catheters [38,39]. The chemical structure of polyurethanes combines hard (isocyanate end-groups) and soft (carbon chains with hydroxyl end-groups) segments, responsible respectively for the mechanical resistance and elastomeric behavior [40]. The unique structure of polyurethanes provides properties such as resistance to abrasion, durability, chemical stability and easy processability. The chemical composition of polyurethanes permits the classification of them as biostable or biodegradable. The last category is incorporated into DDS biomaterials. Polyurethanes are also employed to produce scaffolds for tissue engineering.

As mentioned above, to improve the properties of the polymers (or co-polymers) and add functionalities, their bulky surfaces can be modified to increase, for instance, wettability, biodegradability, bioavailability, cell adhesion, and cell internalization [41,42]. Through organic chemical reactions (amide or ester bonds formation, click chemistry, ring-opening etc.), some moieties can be anchored to the carbon chains making the polymer more hydrophobic or hydrophilic, bioactive, selective etc. The surface of polymers for health applications can be linked to antimicrobials, antioxidants, and/or cell surface receptors (also known as a ligand, exemplified by growth factors, neurotransmitters etc.). Polymer chains can also be attached to inorganic polymers as polysiloxanes.

## 3. Properties of Nanoparticles to Attend Pharmaceutical and Medicinal Applications

The search for more effective therapies than the traditional ones requires multidisciplinary studies in order to develop drugs with less toxicity and greater selectivity and/or pharmaceutical formulations that provide bioactive species with greater chemical stability and solubility. Moreover, administration in smaller doses and modified release are welcomed attributes to reduce side effects. With the advent of nanoscience and nanotechnology, pharmaceutical formulations are increasingly focusing on systems of organic or inorganic nature for the transport or loading of bioactive species that serve the area of nanomedicine. Nanomedicine involves materials on a submicrometer scale, smaller than the dimensions of cells, which have medicinal effects resulting from their unique physicochemical properties in relation to their analogues with larger dimensions (not in the nanometer scale) [43]. These nanoparticles can be used not only in therapies but also in laboratory or imaging diagnosis. 

**Figure 4:**
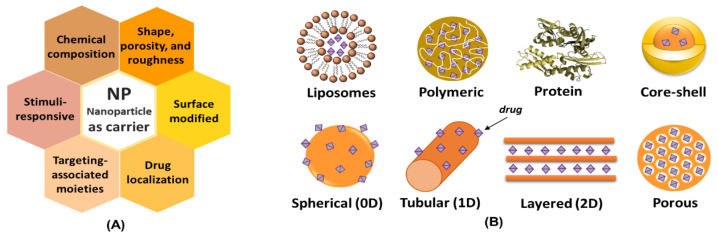
(**A**) Variable chemical and physical parameters considered in the design of the nanoparticles; (**B**) Nanoparticles can have different chemical natures, shapes, porosity, dimensionality etc.

Several types of particles of nanometric dimensions for therapeutic purposes can be engineered to achieve the desired performance in the body. As Figure 4A illustrates, carriers with different physicochemical characteristics are possible, such as chemical composition, size, shape, and porosity, as well as intrinsic surface properties or those modified by functionalization (hydrophobic or hydrophilic, neutral, or electrically charged etc.) [44]. Such characteristics influence biocompatibility, transport, biodistribution, modulation of the immune response and recognition of the nanomaterial by specific sites in the live organism. The design of nanometric carriers also allows for promoting the release of bioactive species through internal or external stimuli, as mentioned before in this text [45]. Thus, nanomedicine can be considered as the application area of nanotechnology in health and medicine. 

Despite only a few decades of existence, nanomedicine has impacted the health area with approximately 200 products (approved or under clinical investigation), which include therapeutic species, agents for in vitro or imaging diagnosis, and medical devices. The first “nano-formulation” approved by the FDA in 1995 is known as Doxil™, a liposome-based formulation as an antitumor drug carrier [46]. Currently, formulations containing drugs inserted in nanoparticulate micelles of the biodegradable poly(ethylene glycol)-poly(D,L-lactide) co-polymer (Genexol-PM™) are also approved. Still, regarding polymeric micelles as carriers, cisplatin encapsulation in a formulation denominated Nanoplatin™ demonstrated its effectiveness in reducing side effects such as nephro- and neurotoxicity. Among the inorganic nanoparticles, the superparamagnetic iron oxide coated with dextran (called Sienna+) was released by the European Commission in 2011 for local administration in the diagnosis of breast cancer by sentinel lymph node imaging [46].

Figure 4B shows examples of organic carriers such as liposomes, polymers, and proteins, together with carriers of spherical (metal gold), tubular (carbon nanotubes), or layered (clay minerals) structures. In some particles, drugs are attached to the external surface, while in other ones, therapeutic species are in porous vehicles or in structures designed *core-shell*, in which one phase is recovered by another, and the drugs can be dispersed in one of them or in both, promoting dual kinetic release profile. Hence, the options of nanocarriers are vast, and their design can be chosen to adjust to the administration route and therapeutic goal.

In the process of intravenous administration of non-particulate drug carriers, one of the biggest problems is the aggregation that occurs in a biological environment. Thus, a major challenge is to find methodologies for obtaining nanocarriers with a low degree of aggregation. Recently, the possibility of using the corona effect of proteins in the stabilization of nanoparticle dispersions in the body has been explored [47]. Studies with albumin and LDH intercalated with chloride, heparin, methotrexate (MTX) or dodecylsulfate ions concluded that this protein has the potential capacity to keep LDHs disaggregated in conditions close to those of the biological environment for the transport and internalization of drugs confined in this inorganic carrier [48,49,50].

Aiming to prepare nanocomposites, the nanoscopic dimensions of the particles promote a huge interfacial region between them and the polymer, consequently fostering uncommon physical, chemical, and mechanical properties when compared to conventional materials in which microscopic particles are dispersed in the polymer [51]. As mentioned forward, the surface of nanoparticles can be modified to improve its compatibility with the polymeric phase, avoiding aggregates of nanoparticles in the organic phase. Hence, the homogeneous dispersion of nanoparticles within the organic phase plays a pivotal role in the preparation of nanocomposites.

## 4. Preparation of Polymer/Nanoparticles (Nano)Composites to Attend Pharmaceutical and Medicinal Applications

Polymer nanocomposites have significant potential in the treatment of cancer and diabetes, among other diseases, and in their diagnosis [52]. The preparation of polymer nanocomposites involves different methods in which characteristics and setting parameters impact the performance of the materials, for instance, mechanical properties, chemical decomposition, gas permeability, the release rate of therapeutic agents, and cell adhesion [52]. Basically, the methods are divided into two approaches: (i) one phase is produced in the presence of the other one (such as the polymerization of the organic monomers in a media containing the other phase) [53], or both phases are produced concomitantly (in situ composite formation) [54], and (ii) the phases are produced separately and mixed (ex situ composite formation) [55], using methodologies suitable to specific characteristics of each phase (solubility, thermal stability etc.) and the looked-for morphological feature. Some polymers can also be produced by microorganisms, i.e., a biosynthetic process, in the presence of the other phase as bacterial cellulose in a medium with graphene oxide [56].

**Figure 5:**
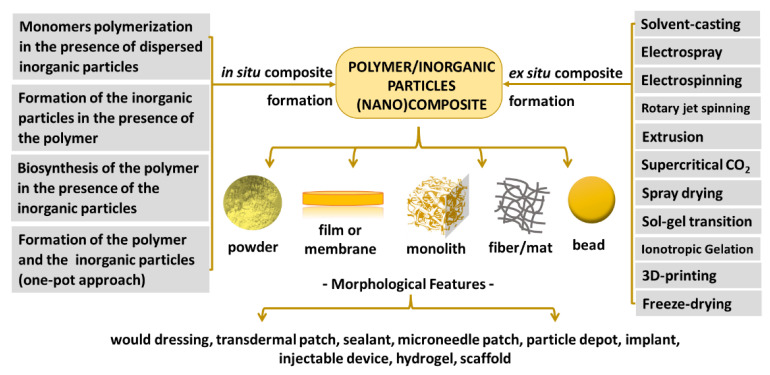
Experimental approaches to produce nanocomposites based on organic polymer and inorganic nanofillers: in situ methods (left side) and ex situ methods (right side).

Considering the biomaterials highlighted in this work, the discussion will focus on the preparation of nanocomposites based on organic polymer and inorganic nanoparticles (nanofillers), which main methods are pointed out in Figure 5, as well as the shapes or forms of nanocomposites that guided to different morphological features. Several techniques are promising for the preparation of nanocomposites comprising inorganic nanoparticles and organic polymers, such as solvent casting, electrospinning, electrospray, supercritical CO_2_, spray drying, sol-gel transition, ionotropic gelation, 3D-printing, freeze-drying. These methods are briefly described ahead.

The solvent evaporation or casting method [57,58] consists of a simple and cheap technique for membrane preparation and is an alternative to more complex methods, such as electrospinning or extrusion, for instance. By this method, a polymer solution or suspension is spread on a substrate. After solvent evaporation under ambient or controlled pressure, humidity, and temperature conditions, polymer precipitates, generating the polymeric membrane. The casting method has been widely employed for the preparation of a variety of polymer/LDH composite membranes from natural or synthetic polymers [59,60,61,62,63,64,65].

Electrospraying and electrospinning techniques allow particle and fiber deposition, respectively, which can be done by means of the same experimental apparatus. Both techniques are based on the application of a high voltage electrostatic field charging a particle’s suspension or the surface of a polymer solution or suspension, respectively, pumped through a small needle leading to the ejection of the liquid jet towards a conductive collector [66]. During the electrospraying process, the solvent evaporates, and the electrospray particles can homogeneously cover the surfaces. Concerning electrospinning, when a strong electric field is established between the metallic needle and the collector, the droplet exiting the needle can take the shape of the so-called *Taylor cone*, from which the charged jet is propelled, elongates, and solidifies; a nonwoven mat is then formed by the dried fiber, which usually has an average diameter ranging from tens of nanometers to a few micrometers [67]. Operational conditions such as the tension and the distance between the needle and the collector, the average molecular weight of the polymer and concentration, the viscosity of the solution, and the vapor pressure of the solvent have to be adjusted to allow proper *Taylor cone* and subsequently fibers formation [68].

Electrospun membranes find applications in diverse areas, such as the food industry and agriculture [69], electrochemistry for energy storage [70], and medicinal device development. In this last field, wound healing purpose has to be highlighted because electrospun nanofibrous mats mimic the extracellular matrix (ECM) components and enable cell growth and differentiation [71]. Fibers possess a high surface area/volume ratio and high porosity, able to create nutrient gradients [68]. Thus, the polymer matrix by itself is promising to be applied as wound dressings or implantable devices, supporting damaged tissues and improving tissue regeneration. Polymer/LDH electrospun membranes are usually prepared via the introduction of particles inside the polymeric fibers. This design is obtained by suspending LDH in the polymer solution, which is further electrospun [72]. Such an approach frequently leads to the formation of beads by increasing the LDH percentage, turning fibers less homogeneous. Alternatively, LDH electrospraying can be combined with polymer electrospinning allowing higher content of LDH particles [73].

Likewise described to electrospinning, the rotary jet spinning method produces centrifugal spun fibers. In the center of a circular structure, a polymer solution or melt polymer passes through the nozzle of a rotary spinneret whose fly towards vertical collector bars, positioned at the border of the circular structure, conducts the deposition of fibers [74]. This technique allows the building of hollow 3D structures that could mimic body cavities, for instance. As an advantage, rotary jet spinning industrial scaling is feasible.

Extrusion consists of applying shear stress in a material at room temperature or under heating (hot-melt extrusion), polymer or polymer suspension, and passing through a fixed cross-sectional profile. Due to the pressure employed in the process, the stability of the formulation is critical and must be controlled to avoid component degradation. Hot-melt extrusion is a technique widely employed in the pharmaceutical field for the production of polymer DDSs (for instance, polyurethanes and Eudragit^®^) covering several administration routes [37,75]. It is needed to be aware that the applied temperature can modify the bioactive species, compromising its bioactivity. Considering that LDH particles are great candidates as nanofiller, as reported in the literature [76,77], its application in the preparation of biomaterials based on nanocomposites by extrusion is suitable. 

Supercritical CO_2_ is an alternative method to replace organic solvents used as reaction media, processing, and dispersant agents by using eco-friendly CO_2_. Supercritical fluids are, in general, obtained by employing a temperature and pressure above the thermodynamic critical point of a substance. Supercritical substances show solvation diffusivity properties, which can be tuned by adjusting pressure and temperature. Usually, CO_2_ is the ideal substance for supercritical solvents and reaction medium in polymerization reactions, since it is less toxic than organic solvents and does not present oxidant properties such as water [78]. Supercritical CO_2_ was promising for surface-modification processes due to its low viscosity, inert behavior, and absence of superficial tension for the development of structure nanomaterials and was already employed for the preparation of polymer/LDH foam composite [79].

Spray-drying allows the preparation of uniform spherical particles and capsules with a rapid processing time [80]. In this method, a precursor liquid is conducted to a piece of atomizer equipment for aerosol formation through a temperature-assisted evaporation process. Then, droplets are directed to a conversion chamber via a carrier gas flow, where droplets undergo evaporation and solute condensation, forming porous particles. LDH particles can be directly atomized or previously combined with a polymer [81,82].

Sol-gel transition can be applied for LDH thin film formation and also for the preparation of polymer/LDH composites [83]. In this method, a colloidal dispersion acquires a gel state through hydrolysis followed by condensation [84]. Hydrolysis can be described by Equation (1), in which the alkoxide group bonded to a metal (usually a transition metal) is cleaved and substituted by the-OH group. After condensation (Equation (2)), O-M-O bounds are formed. M^x+^(OR)_x_ species can be employed for the synthesis of LDH particles with narrow particle size distribution and small size. Residual alkoxide groups could potentially participate in polymer synthesis or curing, allowing great compatibility of LDH-charged particles with hydrophobic polymers.

M(OR)_4_ + H_2_O → HO-M(OR)_3_ + ROH (1)

(OR)_3_M-OH + HO-M(OR)_3_ → (OR)_3_M-O-M(OR)_3_ + H_2_O (2)

Ionotropic gelation is a technique for the preparation of nano or microparticles from the electrostatic interaction between two ionic species in an aqueous medium and under certain conditions in which at least one of the species is a polymer. Polymers such as CS, alginate, collagen (COL), gelatin, hyaluronic acid (HA), and well as mixtures of polymers have been studied. In this sense, small charged molecules and/or ions conduct to the gel state and get effectively encapsulated in the polymeric matrix, which is particularly interesting in the case of drug immobilization [85]. Subsequently, a drying process can be applied, resulting in a powdered product. LDH particles may be added to the polymer solution being entrapped through gel formation [86].

**Figure 6:**
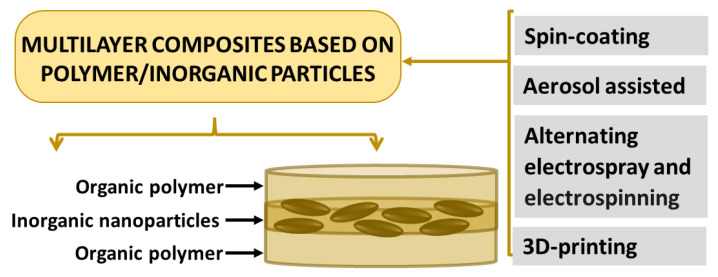
Multilayer composites are obtained by methods that alternate de phase’s deposition to form an object.

3D-printing is a broad and versatile technique used for the manufacture of polymeric materials, but it is not restricted to them; it is also used for the preparation of ceramic and metallic materials. It consists in building objects from the deposition of superimposed layers, as illustrated in Figure 6, based on a three-dimensional model created on a computer. The technique stands out for the diversity of shapes that can be printed, including porous structures whose types of pores and porosity can be controlled, along with the possibility of customizing the materials, pondering the application site, and the function to be performed [87]. When biological species such as cells, growth factors, DNA, or biomaterials (i.e., species with bioactivity) are employed for the object’s preparation, the term *bio-printing* is used. 3D-printing can be used to coordinately deposit LDH particles in the constructive layers of the objects [88], or LDH particles can be embedded into a polymeric ink [89].

Freeze-drying is a process where a frozen solution or suspension is submitted to a low-pressure condition, causing solvent(s) removal through sublimation leading to the formation of porous structures, promising for health and tissue engineering [90]. The porous size is influenced by the freezing temperature. The technique allows the preparation of polymer/inorganic particle composites from water- or organic solvent-based suspensions. Especially for water-based suspensions, a dispersant is necessary to stabilize particles. Emulsions have also been applied as templates to prepare porous material by the polymerization of monomers present in the continuous phase with subsequent removal of the solvent from droplets. LDH and other inorganic particles can be added to a polymer solution prior to freeze-drying originating polymer/LDH composites [91,92]. This technique also allows the preparation of polymer nanofibers from diluted polymer aqueous solutions; then, fibers can act as templates for the preparation of hollow microtubes from inorganic particles [90].

As mentioned above, some techniques allow depositing layers with different chemical compositions and/or phases. Besides electrospraying (associated or not with electrospinning) and 3D-printing, spin-coating [93] and aerosol-assisted [94] methods promote the development of multilayer composites, as illustrated in Figure 6. These categories of processing permit better control of the materials’ properties since different polymers can be used, as well as nanoparticles with distinct functionalities. Consequently, the surface can allocate a drug for fast release, whereas the adjacent internal layers can have a therapeutic payload for long-term release. The design of multilayer composites is flexible and very valuable for the development of biomaterials based on nanocomposites.

The characterization of polymer/inorganic particles nanocomposites involves several techniques but mostly wide-angle X-ray diffractometry (XRD), small-angle X-ray scattering (SAXS), scanning electron microscopy (SEM), transmission electron microscopy (TEM), energy-dispersive X-ray spectroscopy (EDS or EDX), atomic force microscopy (AFM), microcomputed tomography (micro-CT) imaging, thermogravimetric analysis (TGA), differential scanning calorimetry (DSC), vibrational infrared (IR) and Raman spectroscopies, ultraviolet-visible (UV-VIS) spectroscopy (absorbance and transmittance), solid-state nuclear magnetic resonance (NMR) spectroscopy, X-ray photoelectron spectroscopy (XPS), measurements of mechanical properties (tensile strength, Young’s modulus, toughness, flexibility, viscoelasticity etc.), water contact angle, swelling assessment, and gas barrier behavior. Other techniques are used in less extension because it depends on the nature of the individual phases, for instance, Mössbauer spectroscopy and electron paramagnetic resonance. Considering the usage of nanocomposites in pharmacy and medicine, other characterization assays should be selected to attest to their biocompatibility and therapeutic effectiveness.

## 5. Layered Double Hydroxides: Structure, Physicochemical Properties, Biocompatibility

LDHs comprise a class of natural and synthetic materials characterized by a layered structure (two-dimensional/2D organized materials) that can sandwich some species between the layers. Hence, this class of materials belongs to the huge group of intercalation compounds, jointly with graphite, clay minerals, and transition metal dichalcogenides, which interlayer nanospace confines a sort of guest species (from molecules to nanoparticles or biopolymers) depending on the chemical nature of the 2D material [95]. The intercalation process enhances the properties of the host and/or of the confined guest (e.g., (photo)chemical stability or mechanical strength) as well as may engender new properties (electrical conductivity, for instance). LDHs, in particular, have positively charged layers that are neutralized by the presence of negatively charged interlayer ions, and the general formula [M^2+^_(1−*x*)_M^3+^*_x_*(OH)_2_](A*^n^*^−^)*_x/n_*·zH_2_O (M is a metal ion and A is an ion with charge *n*; water molecules in the interlayer space establish a net of hydrogen bonds among them, the hydroxylated layers and the intercalated anions) [96].

Minerals of the following ideal composition were identified among others: [Mg_6_Al_2_(OH)_16_]CO_3_·4H_2_O (hydrotalcite), [Mg_6_Fe_2_(OH)_16_]CO_3_·4H_2_O (pyroaurite), [Ni_6_Al_2_(OH)_16_]CO_3_·4H_2_O (takovite), [Zn_4_Al_2_(OH)_12_]CO_3_·3H_2_O (zaccagnaite), and [Ca_4_Al_2_(OH)_12_]SO_4_·6H_2_O (kuzelite) [97]. LDHs are also known as hydrotalcite-like compounds and are usually abbreviated as M^2+^_R_M^3+^-A, where R is the molar ratio between the di- and trivalent cations. Synthetic analogous can be prepared with high chemical purity and as a single crystallographic phase (often if 0.20 < *x* < 0.33 or R = 2, 3 or 4) by diverse methods, some of them of low-cost and under mild conditions (water medium at room temperature and pressure), as reported in the literature [13,98,99,100].

**Figure 7:**
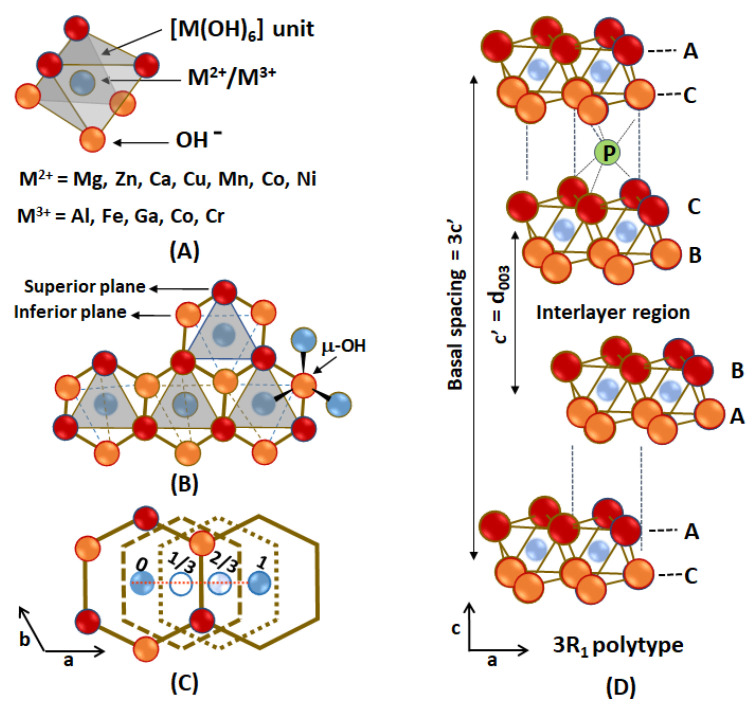
LDH structural aspects: (**A**) [M(OH)_6_] units, (**B**) edge-sharing among octahedra, (**C**) sequence of the layers stacking and (**D**) face-to-face arrangement of the layers. Uppercase symbols A, B, and C represent hydroxide ion positions; P indicates a prismatic site.

The LDH layers are formed by [M(OH)_6_] units containing hydroxide ions in the vertices coordinated to M^2+^ or M^3+^ cations in their centers (Figure 7A). The edge-sharing among octahedra forms layers in which each hydroxide anion is coordinated to three metal cations as a bridging ligand (μ_3_-OH) (Figure 7B) [97]. Each layer can be seen as a closed-packed array of two sheets of hydroxide ions with M^n+^ cations filling all octahedral sites. Considering the plane of edge-sharing [M(OH)_6_] units, the sequence of the layers stacking (a face-to-face arrangement) is not the same, yielding several polytypes [101]. Usually, synthesized LDHs present a polytype with three layers that are related to one another by translations of 1/3 and 2/3 in the a,b plane, as shown in Figure 7C. Hence, the unit cell of such polytype has three layers (rhombohedral structure) or three interlayer regions, and the eclipsed arrangement of hydroxide ions in adjacent layers creates prismatic sites in the interlayer region (Figure 7D). The basal spacing of LDHs is determined by the size of the intercalated payload, their negative electric charge, and the arrangement assumed in the nanospace (for instance, parallel, perpendicular, or tilted arrangement of guests in relation to the layers; monolayers, bilayers, multilayers, or interdigitated organization of guest species).

The small thickness value of the LDH layer (less than 0.5 nm) confers to this material the classification as a nanomaterial, defined by the European Union as "*a natural, incidental or manufactured material containing particles, in an unbound state or as an aggregate or as an agglomerate and where, for 50% or more of the particles in the number size distribution, one or more external dimensions is in size range 1–100 nm*” [102]. The divalent and trivalent metal cations that form the layers have crystal ionic radii close to the values for magnesium (0.0860 nm) and aluminum (0.0675 nm) values to fit in the octahedral hole, stabilizing the layer structure [13]. The isolation of LDH having three (ternary) or four (quaternary) different cations is also possible, which opens the opportunity to insert endogenous bioactive metal cations in safe amounts in the intralayer sites (e.g., antimicrobial gallium ion) or doping it with a cation appropriate for diagnosis by imaging (e.g., paramagnetic gadolinium ion).

**Figure 8:**
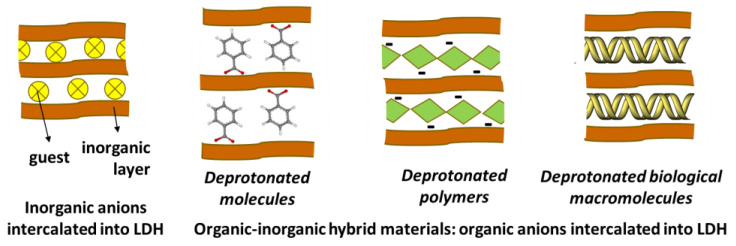
LDH materials can be intercalated with inorganic or organic anions to neutralize the layer’s charge.

The anions intercalated into LDH can be simple inorganic ions (carbonate, chloride, sulfate, nitrate etc.) [96], organic ions (terephthalate, dodecylsulfate etc.) [103,104], polymers (*carboxymethylcellulose* (CMC), alginate etc.) [105,106], drugs (anti-inflammatories, anticancer drugs, antibiotics, antihypertensive, anti-diabetes etc.) [22,24], vitamins (vitamin C, folic etc.) [107,108], phytochemicals (curcumin, coumaric, abietic, norbixin) [109,110,111,112], and nucleotides (DNA and RNA) [14,15,16,17,18,19,20,21,22,23,24,25], as represented in Figure 8**.** Hence, by combining different metal cations, stoichiometries and intercalated species, a huge number of LDH materials can be engendered to attend to pharmaceutical and medical needs.

LDHs are synthesized by different methods categorized as direct or indirect methods [13,15,96,98,99]. Direct synthesis methods are also known as one-step methods, for example, coprecipitation, while indirect synthesis methods involve more than onestep, such as anion exchange, anion exchange by regeneration of calcined material, and exfoliation-reassembling process. Most studies have used the coprecipitation or ion exchange method. The first method permits the formation of hydroxylated layers in the presence of the species to be intercalated, as shown in Equation (3) for the preparation of hydrotalcite:

(3) $$6{\mathrm{Mg}}_{\left(\mathrm{aq}\right)}^{2+}+2{\mathrm{Al}}_{\left(\mathrm{aq}\right)}^{3+}+16{\mathrm{OH}}_{\left(\mathrm{aq}\right)}^{-}+{\mathrm{CO}}_{3\left(\mathrm{aq}\right)}^{2-}\to \left[{\mathrm{Mg}}_{6}{\mathrm{Al}}_{2}{\left(\mathrm{OH}\right)}_{16}\right]{\mathrm{CO}}_{3\left(s\right)}$$

Coprecipitation is a self-assembling process, and organic anion can affect the morphology and size of the hybrid particles, for instance, the drug sulindac, attested by electron microscopic techniques and XRD data [113]. Usually, magnesium, zinc and aluminum are the cations chosen for DDS preparation, and Mg_R_Al-LDH presents small particles and less crystalline materials than the analogous Zn_R_Al-LDH independent of the inorganic or organic nature of the anion in the synthetic medium to be intercalated [114].

Materials 2D can be exfoliated, i.e., can undergo a “*process by which the layers of a multi-layered structure separate*” [4] and this property is useful to prepare a colloidal dispersion of anisotropic nanoparticles for intravenous injection or to obtain nanoparticles dispersed in polymer nanocomposites for other routes of administration. LDH materials can be exfoliated in organic (DMF, formamide, butanol and xylene) or water solvent under ultrasonic treatment or mechanical stirring [115,116,117]. The exfoliation in water requires the presence of intercalated species (e.g., formate, acetate, and lactate) to swell the interlayer space and promote the separation of the layers. The exfoliated dispersion in a solvent can be mixed with a solubilized polymer or with its monomer followed by the polymerization process. Additionally, the nanocomposite can be achieved by melting the polymer if the thermal stability of LDH allows it.

**Figure 9:**
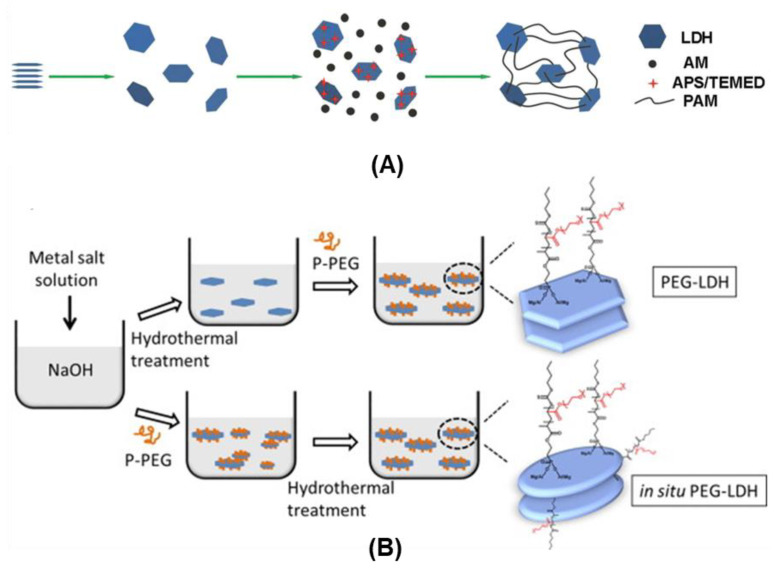
Schematic representation of methods used for (**A**) preparation of PAM/Mg_R_Al-LDH hydrogel by in situ polymerization; AM: acrylamide; APS: ammonium peroxydisulfate; TEMED: *N,N,N′,N′*-tetramethyl-ethylenediamine (reprinted from Wu and Chen [120] with permission of Wiley-VCH GmbH) and (**B**) PEGylation of LDH nanolayers (reprinted from Cao et al. [121] with permission of Elsevier Inc.).

Nanocomposites films of PVA/LDH [118] or PCL/LDH [119], both biocompatible and biodegradable polymers, were prepared, respectively, from a dispersion of the inorganic particles in water and from the dispersion of the inorganic phase into PCL by a high energy ball milling. In another example, polyacrylamide(PAM)/Mg_R_Al-LDH hydrogel was prepared by the exfoliation of Mg_R_Al-isethionate in water with posterior in situ polymerization of acrylamide in the presence of an initiator and an accelerator [120], as represented in Figure 9A.

Single layers are suitable for polymer/LDH nanocomposite preparation, but they can be obtained by routes distinct from exfoliation. Yu et al. [122] reported a “bottom-up" method to produce single layers of LDH with plate-like morphology, with average lateral size ranging between 25–50 nm and thickness of 0.8 nm, noticed by HRTEM and AFM techniques, respectively. The method consists of a one-step synthesis performed directly in a mixture of water and formamide. Such a method has been used to load diverse BPs with therapeutic and/or diagnostic properties for cancer treatment [123,124,125,126,127].

Stable and monodispersed LDH nanolayers can be obtained by separating the nucleation and growth particle steps using a flash coprecipitation synthesis method that consists of a fast mixing of NaOH and metal salt solutions followed by hydrothermal treatment, reported by Xu et al. [128]. The preparation of monodispersed Mg_3_Al-LDH material hosting radioisotopes (^64^Cu^2+^ and ^44^Sc^3+^; PET imaging agents) [129], Mn^2+^ (T_1_-MRI payload) [130] or Cu^2+^ (T1-MRI and photothermic payload) [131] in the intralayer region were also reported. The functional metal cations can be hosted by partial isomorphic substitution of cations in the LDH layers in a second synthesis step. Mn_x_Mg_3−x_Al-LDH, for instance, presented an average hydrodynamic diameter of 48.0 ± 1.8 nm (20–80 nm) and an average thickness of 13.7 nm.

The flash coprecipitation method was used to prepare hybrid materials by interacting the monodispersed LDH nanolayers with drugs/imaging agents [48,131]. In addition, bovine serum albumin (BSA)/LDH nanocomposites were prepared by covering the surface of nanolayers with BSA [48,129,130,131], which can be further crosslinked in the LDH surface and/or posteriorly modified with targeting and imaging agents [49,132]. Cao et al. [121] reported the PEGylation of nanolayers by adding phosphonic acid-terminated PEG before or after the hydrothermal step (Figure 9B). The addition of PEG before the hydrothermal treatment aimed to obtain exfoliated and biodegradable PEGylated Fe_2_Al-LDH with catalytic Fenton properties (ROS generation) for cancer therapy [133]. The PEGylation and corona effect promoted by BSA covering confer stability to LDH nanoparticles in biological media and increase the half-time of materials in the bloodstream by hindering aggregation due to nonspecific protein absorption, which can improve their bioavailability and tumor cell uptake.

LDHs show some characteristics of ceramic materials applied in medicine and dentistry since they are inorganic materials that exhibit a combination of ionic and covalent bonds. However, LDHs do not have high melting points (indeed they undergo dehydroxylation at about 200–300 °C) or high hardness such as Al_2_O_3_, ZrO_2_, TiO_2_, Ca_3_(PO_4_)_2_] or silica-based glasses, which are examples of bioceramics [134]. In terms of in vivo reactivity, LDHs have a favorable balance between chemical stability and biodegradability, reminding bioactive bioceramics such as silica-based glasses and glass-ceramics comprising mostly the chemical elements silicon calcium, sodium, and phosphorus [134]. The reactivity of bioceramics depends on their chemical composition, particles mean size, level of crystallinity, porosity, and defects, as observed for LDH materials, allowing to adjust of the rate of materials solubilization. 

The biological environment where the biomaterial should operate needs to be considered, along with the organism pathways to be crossed in certain modes of administration. Biological fluids have anions, cations, several proteins, and dissolved gases (O_2_, CO_2_ and N_2_) at 37 °C and pH at about 7.4, which can change the surface of the material and provoke solubilization [134]. The biomolecules can increase the dissolution process if stable metal complexes are formed, shifting the equilibrium towards the soluble species. Metal cations such as iron, zinc and copper are not free in the organism but coordinated with the biomolecules, which affect metal transport, cell uptake, clearance etc.

In the case of LDH, solubilization promoted by the neutralization of hydroxide layers releases metal cations in a specific local of the organism (gastrointestinal system, blood, eyes, skin, bone etc.). Hence, LDH has intrinsic biological active properties driven by the identity of the metal cation, its concentration (that can be modulated by the solubilization rate), and its form in the environment, i.e., the metal speciation. In this way, the chemical composition of LDH layers can be chosen to attend to the therapeutic goal. The alkaline nature of LDH also can contribute to enhancing the biological response, such as for instance, osteoblast proliferation in bone regeneration [135].

**Figure 10:**
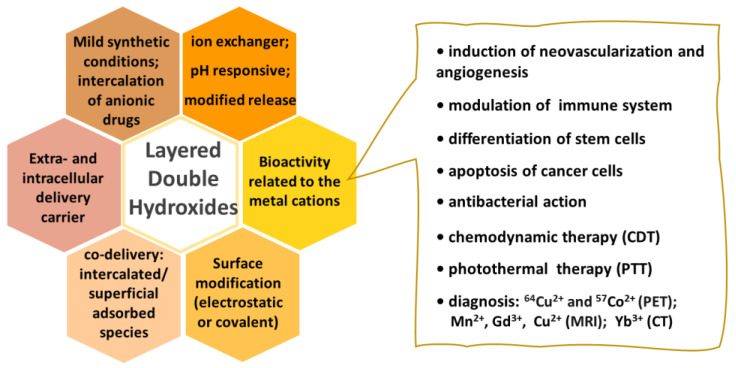
Suitable LDH properties for therapeutic and/or diagnosis purposes.

Synthetic LDH having the composition of the mineral hydrotalcite, [Mg_6_Al_2_(OH)_16_]CO_3_·4H_2_O, is commercialized as an antacid with the brand names Talcid™ and Gastrum Plux™, among others [136]. Studies carried out in 2000 showed that the hydrotalcite-like compound is not just an antacid, but it can protect the gastric mucosa, activates angiogenesis in injured mucosa, and accelerates human gastric ulcer healing [137,138]. Such bioactivity is related to the activation of prostaglandin synthesis and the activation of genes for an epidermal growth factor (EGF) and its receptor in normal and ulcerated gastric mucosa. Years later, investigations about the hydrotalcite-like compounds as suitable carriers for DDS development motivated studies about their intrinsic bioactivity. Figure 10 highlights the LDH properties that induce studies comprising the application of this class of intercalation compounds in health-related concerns. 

Some appropriated physicochemical characteristics of LDH to be recognized as a potential biomaterial were introduced later. Besides the modulation of injured tissue repair [138], LDH without any immobilized drug (i.e., intercalated with simple anions like carbonate and chloride) can trigger apoptosis of cancer cells by regulation of gene expression [139], shows immunomodulating activity [140], promotes osteogenic differentiation [140,141], or induces neovascularization and angiogenesis [142,143]. LDH layers can be doped with radioisotopes for positron emission tomography (PET) [129,144], with paramagnetic cations for magnetic resonance imaging (MRI) [125], or with ytterbium for X-ray computerized tomography imaging (CT) [127].

Regarding DDS materials, one parameter to be highlighted is its loading capacity (LC), which value is usually lower than 10% [145]. The LC of LDH compounds is related to the amount of trivalent ion in the layers (the *x* value in the general formula [M^2+^_(1-*x*)_M^3+^*_x_*(OH)_2_](A*^n^*^−^)*_x/n_*), and with the electric charge of intercalated anions (the *n* value in the general formula). Considering both parameters, loading capacities of 30–50% can be reached [110,111,146]. However, the size/area of the anion can preclude the total neutralization of positively charged layers because of steric hindrance, decreasing the LC, as observed for LDH-pravastatin material [146]. The intercalation method (coprecipitation or ion exchange, for instance) can also influence drug loading, as noticed in studies with naproxen [147]. Another kind of interaction can be established beyond the electrostatic one between positively and negatively charged species, as reported when using ultrathin LDH particles in the presence of doxorubicin; for example, LC was 344.56% [126].

The structural M^n+^ ions allocated in the hydroxylated layers can add functionality to LDH as follow: zinc, magnesium, and iron ions, natural elements in bone tissue, can be incorporated in scaffolds for bone tissue engineering [135]; copper and zinc for antimicrobial therapies concerning skin wound healing [148,149]; aluminum in vaccine carriers [150]; iron, copper, or manganese biomaterials for Fenton-like catalytic reactions applied in chemodynamic therapy (CDT), endogenously activated, or photodynamic therapy (PDT) and sonodynamic therapy (SDT), endogenously activated [151].

**Figure 11:**
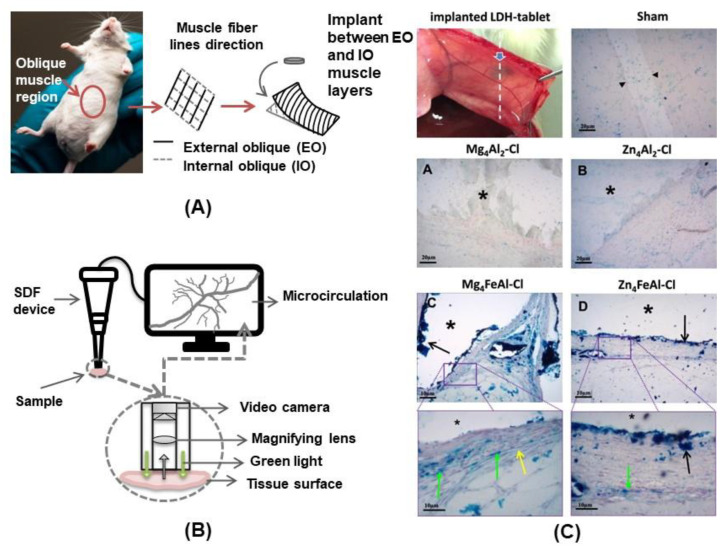
Schematic illustration of (**A**) LDH tablet implanted in the intramuscular abdominal region of rats and (**B**) microcirculation monitoring by SDF videomicroscopy image (both figures reprinted from Constantino et al. [152] with permission of World Scientific Publishing Co.); (**C**) Iron histochemistry findings following A-Mg_4_Al_2_-Cl LDH, B-Zn_4_Al_2_-Cl LDH, C-Mg_4_FeAl-Cl LDH and D-Zn_4_FeAl-Cl LDH tablets implants between abdominal wall intermuscular spaces, after 28th P.O. The white dotted line with the blue arrow indicates the macroscopic appearance of the implanted LDH tablet and the cut line for histological processing. LDH tablet (*); positive staining (→ arrow black); positive staining in fibroblast (→ arrow green); positive staining in ECM matrix (→ yellow); perimysium between abdominal wall muscle layers (►). Prussian blue staining (reprinted from Figueiredo et al. [143] with permission of American Chemical Society).

The biocompatibility of LDH materials has been investigated by a set of in vitro (cell viability, hemolysis, ROS generation etc.) and, in a lesser amount of studies, by in vivo techniques [152]. The innate biological response of LDH was evaluated by the tablets implantation in the rat abdominal intramuscular wall and, for the first time, an assay based on the microcirculation to investigate the biocompatibility of such kind of material [142]. LDH tablets of composition M_2_Al-Cl and M_4_FeAl-Cl (M^2+^ = Mg or Zn) were implanted as illustrated in Figure 11A, and the peripheral microcirculation in real time was monitored by side-stream dark field (SDF) videomicroscopy, a non-invasive imaging tool (Figure 11B), after 28 days of implantation, in addition to the tissue analysis by histopathology (Figure 11C). The visualization of microcirculation around the tablets showed a normal circulatory network aspect, no blood flow obstruction (thrombosis), hemorrhage or signs of tissue inflammation. The formation of a fibrous capsule was not observed. Hence, the materials did not induce an immune response, encouraging their bio-application. The histological analysis revealed endothelial adhesion, fibroblast proliferation, inflammatory response modulation, presence of neovessels and tissue integration, supporting the biocompatible use of such materials for implant purposes.

LDH-drug materials can have a role not only in tissues but also in the intracellular environment. Choy’s research laboratory studied the mechanism of LDH particle internalization into cells and observed an endocytosis process, intermediated mainly by the protein clathrin, which is time, dose and particle size-dependent [153,154]. The cell encloses the inorganic particles in a vesicle made from a plasma membrane. LDH particles are accumulated in the endosomes, where can be partially dissolved and/or undergo ion exchange reaction because of the presence of ionic species (H^+^ and Cl^−^) [155]. LDH can go out of the endosomes into the cytoplasm, where an intercalated ion can be delivered by exchange with anionic species in the environment. Particles in the cytosol with less than about 35 nm in size can pass through the nuclear membrane reaching the nucleus [156]; this result shows the potential of LDH for gene therapy.

The surface modification of LDH by polymers is an important strategy for their application in the pharmaceutical field owing to the enhancement of key characteristics of these hybrids, such as improved stability, biocompatibility, dispersity, cellular uptake and decrease in systemic toxicity [52,157,158]. The interaction process of organic molecules and the surfaces of LDH particles can involve different regions of the inorganic phase, such as the interlayer space and/or the external surface [159]. However, methodologies involving mainly the interaction of polymers with the outer surface of the LDH phase allow a larger uptake of a certain bioactive compound, which could benefit drug delivery applications, for instance [160]. Such interaction between the LDH surface and a polymer or a therapeutic agent occurs primarily through Coulombic interaction caused by the positively charged layers of the inorganic phase [159,161], which can be optimized when the organic species have negatively charged groups within their structures. However, a large variety of organic molecules are neutral or do not possess significant hydrophilic portions to interact with the hydroxylated surface of LDH, which can cause poor compatibility between both phases [162] and, consequently, worsen several physical properties of the composite. The surface of the layered structure can also be modified with specific moieties (ligands, for instance) that target a given therapeutic or imaging purpose [163,164].

**Figure 12:**
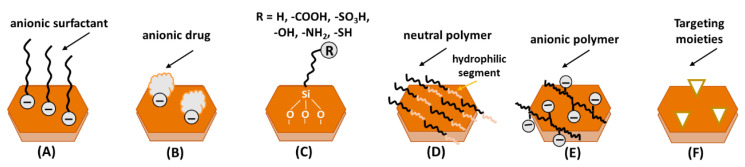
Strategies to modify the LDH surfaces through electrostatic or covalent linkages: (**A**) hydrophobization of surfaces by anionic surfactants; (**B**) hydrophobization of surfaces by anionic drugs having hydrophobic groups; (**C**) surfaces modification by silanization reaction; (**D**) surface modification with neutral polymers having hydrophobic and hydrophilic segments; (**E**) surface modification with charged polymers; and (**F**) surface functionalization with targeting molecules.

One route to improve the overall affinity of both inorganic and organic materials is related to the intercalation of hydrophobic species, such as surfactants and even hydrophobic drugs (Figure 12A,B). This modification can also assist the interaction between the polymer and the interlayer region of LDH, allowing a good dispersion of the inorganic phase over the hydrophobic polymeric matrix and improving their medical applications, as a result in the enhancement of physical properties of these hybrids such as mechanical, thermal and barrier [165]. Further compatibilization between LDH and polymeric phase can be achieved by the chemical modification of the hydroxide units present in the layered structure with different silanol groups, depending on the hydrophilic/hydrophobic characteristics of the macromolecular structure [166] (Figure 12C). The external surface of LDH can be decorated with polymers as PEG, that have hydrophobic and hydrophilic segments (Figure 12D) or charged polymers as CMC (Figure 12E). Therefore, when looking for target delivery, the nanoparticles’ surface can be functionalized with active targeting molecules to reach membrane receptors overexpressed by cancerous cells (Figure 12F). 

**Figure 13:**
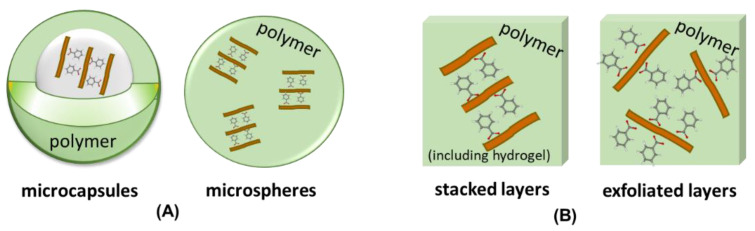
Polymer/LDH nanocomposites: (**A**) microcapsules and microspheres-based delivery systems; (**B**) intercalated and exfoliated nanocomposites.

Once the surfaces of the inorganic layers are modified or functionalized, the polymer/LDH nanocomposite preparation can be achieved by the methods presented in Figure 5 and Figure 6, as microcapsules (membrane-like shell surrounding a core containing the LDH-drug) or microspheres (LDH-drug particles homogeneously dispersed in a polymeric matrix), shown in Figure 13A. The hybrid particles can be dispersed in a polymer and manufactured as wound dressing, transdermal patch, microneedle patch, implant, injectable hydrogel, or scaffold, among other forms. During the processing step, LDH particles can keep the stacking arrangement of the layers, producing an intercalated nanocomposite. On the other hand, layers can be separated, producing an exfoliated nanocomposite. Figure 13B illustrates such classes of polymer/2D filler, which confer to the nanocomposites very distinct physical, mechanical, thermal, and gas barrier properties. Such arrangement of the hybrid filler in the polymeric matrix should also address different release mechanisms and rates.

Table 1 brings the review works published from 2017 up to now about polymer/LDH composites associated or not with other phases. Details about such materials, their properties and applications will be presented in the next items.

The surface modification of LDH particles deserves special attention in the development of theragnostic (from the words *therapo-gnostics*) agents, an expression introduced in 1998 by Funkhouser as the capacity to access the diagnostic and the therapy of a disease in a single step [174]. A theragnostic agent is comprised of a biomedical payload (BP), a carrier, and a surface modifier [175]. The BP comprehends a large spectrum of organic and inorganic species that are specifically responsible for imaging and/or therapy. Several BPs can be intercalated between LDH layers (e.g., isophthalic acid, a photosensitizer) [176] or loaded on particles’ surface (e.g., indocyanine green, a sulfonated dye) [127] or hosted in the layers (e.g., Cu^2+^, both T1-MRI and photothermal agent) [131]. The carrier is responsible for delivering the BPs through the body into the desired local for therapy and diagnosis [175]. Finally, the surface modifiers, as the name suggests, are responsible for conferring steric/electrostatic stabilization and changing the physicochemical properties of the carrier surface, such as surface charge and hydrophobicity [175,177,178].

Surface modifiers stabilize theragnostic nanoparticles in fluid blood by avoiding their aggregation and overcoming the clearance pathways of nanoparticles in the organism, prolonging the circulation time in the bloodstream and/or enable that the material preferentially targets a specific site [175,177,178,179]. Consequently, their biodistribution is improved because the attack from the immunologic system is decreased, and the accumulation in organs of the reticuloendothelial system (liver, spleen, and lung) is reduced, allowing the systemic administration of the theragnostic nanomaterial [175,177,178,179]. The administration of theragnostic materials in routine medical procedures has the potential to achieve: (i) localized therapy, a non-invasive treatment that can decrease side effects, (ii) evaluation of delivery and distribution of therapeutic agents by imaging, (iii) monitoring the treatment efficacy in real-time, and (iv) personalized medicine [175,180]. Although there are some FDA-approved DDSs (e.g., polymers and vesicles) [35,181], most theragnostic materials are still in pre-clinical evaluation, especially those constituted by LDH [172].

Up to now, the in vivo studies involving LDH as a carrier in theragnostic materials reported applications in cancer treatment models. For this application, the theragnostic agent is administered by intravenous [125,126,127,130,131,176] or intratumoral injection [123,124,125,182,183], which require stable dispersions in biological fluids [184]. Besides, small particles (higher than 5 nm and lesser than 200 nm) are needed to prolong bloodcirculation half-time and to avoid the clearance by the reticuloendothelial system, favoring the accumulation of nanoparticles in the tumor tissue by enhanced permeability and retention (EPR) effect [181,185]. At a first glance, LDH seems not to be suitable for theragnostic therapy carried out through systemic administration because of its poor stability in water and physiological fluid in relation to particle aggregation [48,50,184,186]. However, LDH materials show remarkable versatility since it allows the combination of different functional metals in the layer composition (e.g., Cu^2+^, Mn^2+^, rare-earth metals etc.) with diverse intercalated counter anions or loaded molecules [124,125,126,127,130,131,176]. 

The preparation method of LDH can guide the formation of ultrathin particles (or single layers) in the 20–200 nm size range [125,126,127,130,131]. Owing to the plate-like morphology and thin thickness, LDH monolayers possess extremely high surface area, leading to outstanding values of drug loading capacity [126,127], a desired property for the development of theragnostic materials. Organic BPs loaded in LDH monolayers are stabilized and well dispersed, being released at a slow rate in the pH of normal body fluid (pH 7.4) [125,126,127]. Compared to pristine (or commercial) diagnosis and therapeutic agents, BPs loaded into LDH present superior effects in a minor administration dose, as for instance: the low solubility of a hydrophobic drug in physiological media can be overcome, the fast clearance by the organism can be prevented and the side effects can be lowered [125,126,127,130,131,182,183,187]. Furthermore, LDH monolayers present anisotropic properties such as the electron-withdrawing and confinement effects, which can enhance the photodynamic/photothermal properties and photoelectron activities of BP (emission intensity and sensitivity enhancement by avoiding bleaching) [123,124,125,183]. In addition, LDH responds to endogenous pH value, which is an advantage in remembering the acidic microenvironment of the tumor (pH = 6.5–7.0), early endosome (pH 6.0) and late endosome/lysosome in cells (pH 5.0) [130]. The slow solubilization of LDH in acid media promotes drug release in a controlled pathway [125,126,127] and raises the magnetic resonance relaxivity of LDH containing paramagnetic cations by the structural defects generated during the inorganic particle dissolution [130,131].

BSA is a promising surface modifier considering that it is the most abundant protein in human blood [178,186]. Giacomelli and co-workers [50,186], as well as Xu and co-workers [48,49], were pioneers in the BSA-coated LDH studies. Such protein generates a steric effect between the inorganic nanoparticles, stabilizing them in electrolytic solutions and cell culture medium [50,178,186]. Furthermore, BSA-coating can reduce the adsorption of opsonins and inhibit the uptake of nanoparticles by mononuclear phagocytes, extending their half-life in the bloodstream [131]. Because of these outstanding features, BSA-coated LDH nanoparticles are extensively applied in the context of theragnostic materials and/or LDH-administered by intravenous route [129,130,131,132].

Beyond albumin, polymers and co-polymers can also be utilized as LDH surface modifiers for theragnostic purposes in cancer treatment. Yan et al. [188] reported the PEGylation of LDH by PEG-carboxy-conjugated aminopropyltriethoxysilane as it is an alternative for surface modification of LDH nanoparticles, which demonstrated to be suitable for MTX systemic administration. Xu and co-workers [189] reported the use of LDH-coated with pH-sensitive charge-reversible polymers to enhance the blood circulation of theragnostic nanoparticles and, consequently, their passive targeting into the tumor tissue. The use of modified co-polymers that comprise PEG [189] and poly(2-aminoethyl methacrylate hydrochloride) [189] conjugated with dimethyl-maleic acid allowed the selective action of Cu-LDH in the tumor environment.

Active targeting molecules are promising in cancer treatment because cancerous cells overexpressed membrane receptors to specific molecules. One of the most attractive targets is folic acid (or folate) since many cancerous cells overexpress the folate receptor. Park et al. [190] studied passive and active targeting strategies for cancer gene therapy by applying survivin siRNA loaded in LDH nanoparticles. Passive targeting mediated by the EPR effect was undergone by Mg_2_Al-CO_3_ particles with 100 nm of average size and hexagonal shape. On the other hand, the active targeting was evaluated by Mg_2_Al-CO_3_ conjugated with folic acid (LDH-FA). Better results were noticed when handling the LDH-FA hybrid material. Using a similar approach, Wen et al. [191] prepared a carrier constituted by stratified layers of CoAl-LDH and MnO_2_ conjugated with folic acid. More details about the application of LDH as a carrier in theragnostic agents are reported in review works [159,172,192,193,194].

## 6. Drug Delivery Systems Based on Organic Polymers and Layered Double Hydroxides Nanocomposites

Recent developments in the field of biomaterials have facilitated the preparation of alternative therapeutic systems to traditional pharmaceutical forms, resulting in a considerable improvement in drug administration procedures. This is especially important because there are many disadvantages associated with the direct use of certain drugs. Before 1950 drugs were compacted directly like pills and released without any discrimination upon contact with an aqueous medium [195]. However, after the first sustained-release formulation introduced by Beecham in 1952 [196], the recent developments of new and more effective therapeutic systems as an alternative to traditional pharmaceutical forms resulted in a considerable improvement in drug administration techniques. These more advanced systems in their conception and capacity to release the active ingredients are the DDSs, devices that release one or more active principles at predetermined rates and times, depending or not on the environment in which they are applied, following a particular purpose.

Although this modern drug delivery technology is only 70 years old, it has evolved spectacularly to develop the most numerous and attractive DDSs for various diseases and clinical applications. Thus, DDSs have three well-defined phases in their development: (i) the first generation (1950–1980), which was very important in the production of new oral and transdermal formulations of drugs controlled release, showing as its main limitation the physicochemical barriers [197]; (ii) the second generation (1980–2010), focused on “smart drug delivery”, where the release of insulin via the pulmonary route was significantly explored (however, the product was withdrawn from the market due to low bioavailability compared to parenteral administration) [198], and on tumor-targeted drug delivery using nanoparticles, where biological barriers as the main challenge; and (iii) the third generation (from 2010 onwards) which are focused on “modulated delivery systems” and need to overcome both physicochemical and biological barriers [195] and, concomitantly, show a high drug retention capacity and the maintenance of drug concentration in blood or target tissues at the efficacious level (besides low-cost and ease manufacturing using conventional equipment and techniques) [199].

The effectiveness of a DDS depends on the combination of three fundamental factors: (i) specific cellular binding, (ii) intracellular absorption of the nanocarrier by target cells, and (iii) release of the drug in its active form. Among these topics, the release is a critical aspect of the process and, therefore, must occur in a precisely controlled manner for the drug to perform its therapeutic efficacy with good bioavailability in the bloodstream [200]. However, since biological mechanisms occur at the nanometric scale, nanocarriers show increasing interest mainly because they have the ability to interact with specific cellular targets [201]. In this regard, the DDS design is generally based on the physicochemical and pharmacokinetic properties of the drug and how these properties are associated with the material that will play the role of carrier and protector of the active principle.

**Figure 14:**
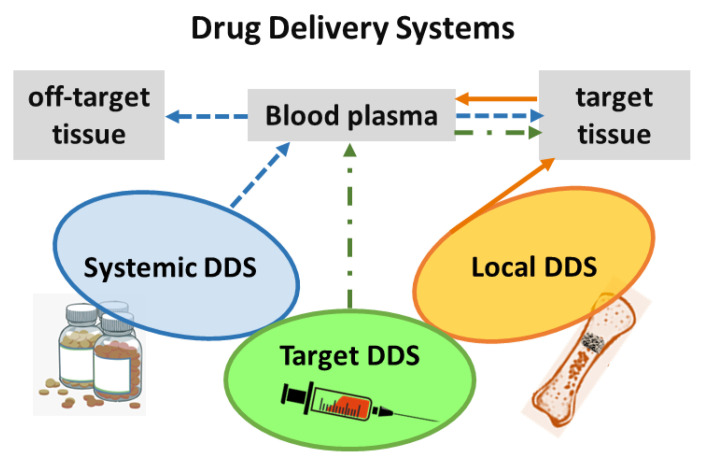
Classification of the Drug Delivery Systems considering the administration route.

DDSs are classified considering the administration route (Figure 14) [26]: (i) systemic drug delivery, in which the drug is released directly (by intravenous injection) or indirectly (by oral pills) in the blood plasma, and transported to the target and off-target tissues; (ii) local drug delivery in the target tissue preventing that the drug reaches off-target tissues; (iii) targeted drug delivery, administrated in the blood plasma to reach a target tissue by the specific design of the DDS.

LDH has been studied as a nanocarrier applicable in DDS for different purposes. For example, LDHs can intercalate bioactive species establishing arrangements and physical interactions that favor solubilization compared to those ones in the pristine drug crystal. Many drugs, such as the anti-inflammatories ibuprofen [202] and zaltoprofen [203], the diuretic piretanide [204], and the vitamin B3 (nicotinic acid) [205], among others, were manipulated by intercalation into LDH to overcome the low solubility challenge. The solubility test of zaltoprofen in water and phosphate buffer (pH equal to 6.8) showed that its intercalation into Zn_R_Al-LDH improved three times its solubility compared to the non-intercalated drug [203] and presented better performance than the physical mixture of zaltoprofen and LDH, demonstrating the benefits of the DDS approach. The therapeutic efficacy of a drug in a disease depends on its bioavailability in the bloodstream. Working just on the structure modification of bioactive molecules is not enough to achieve this purpose [26,206]. Hence, the use of a DDS is a suitable approach to improve the drug action by enhancing its solubility through (i) adsorption on the carrier surface, avoiding the previous crystal building formation or (ii) by the confinement inside the carrier structure to change the intermolecular interactions. These processes improve the drug solubility and its release by diffusion, dissolution, or swelling.

Another motivation for the encapsulation of therapeutic species into a carrier is to improve its shelf life and prevent its decomposition along the pathway process in the organism. Considering the oral administration, the changes in pH values to achieve local absorption can cause the breakage of the drug’s intramolecular bonds and the loss of its action, besides the formation of toxic products [26,206]. Vitamin C is sensitive to light, oxygen, and heat, so the intercalation process can increase its chemical stability. Aisawa et al. [107] evaluated the light and heat resistance of ascorbate ion intercalated into LDH after exposing the samples to a simulator of sunlight for 24 h at 303 K and, in another experiment, to heat (313 K) for 30 days under air. The oxidation rate of the confined vitamin was slower when compared to the free vitamin. Vitamin B3 (nicotinic acid), which is also very sensitive to degradation processes, was protected and presented better thermal stability intercalated into the Zn_R_Al-LDH when compared to the free molecule [205].

DDS strategy can also increase the drug bioavailability. The capacity of LDH to enhance the bioavailability of confined bioactives without causing toxic effects in the bloodstream because of its ability to promote a sustained release was reported in the literature [152,206]. The composite material Mg_R_(Al,Gd)-LDH containing atorvastatin (a neuroprotective drug) and ferritin (a blood –brain barrier transport agent) was prepared to aim the suppression of ischemic stroke–reperfusion injury, as reported by Wang et al. [207]. The hemolytic activity in vivo was relatively low (about 3%; the secure value is 5%) even in a high concentration of the material (100 ppm). This result allowed the composite administration by intravenous injection in mice since it presents an elevated hemocompatibility. Furthermore, the biochemical indicators of the liver functions (such as alanine transaminase and aspartate transaminase) showed no alteration. The route of administration affects the drug bioavailability, which could have contributed to the outstanding performance of the composite: reduction of neurons apoptosis and oxidative damage in the brain cortex.

Several nanocarriers based on organic units and inorganic compounds are developed specifically for the purpose of application in nanomedicine. In this sense, nanocomposite systems resulting from the assembly of polymers that are combined at the molecular level with LDH have achieved prominence in recent years with special emphasis in the biomedical field, such as drug delivery and tissue engineering applications. These nanomaterials can also show a wide range of responses to stimuli, such as pH, temperature, ionic strength, and concentration, becoming particularly attractive for the development of novel, and even smart, oral drug delivery systems, offering new targeting strategies for disease diagnosis and treatment [208].

As an example of a promising nanocomposite system, doxorubicin (DOX), a drug used for a broad spectrum of cancer (breast cancer, bladder cancer, sarcoma etc.), was intercalated into MgAl-LDH with a polyacrylic polymer [209]. Confocal microscopic images showed the improvement of DOX bioavailability because the amount of the drug inserted and maintained into the cell nucleus of human osteosarcoma cells (MG-63) and human lung adenocarcinoma epithelial cells (A549) for the efficacy of the treatment were pronounced compared to free drug In another study, DOX immobilized on LDH-CuAl decorated with poly(phenylenediamine) promoted a sustained release of the drug in pH 7.4 [210]. Moreover, according to confocal laser scanning microscopy, the nanocarrier increased the biocompatibility in normal (PC12 and HEK298) cells and the anticancer potential in HT-29 cells by enhancing the internalization of it into the cells compared to the free drug.

Treatment of ocular diseases is challenging due to the difficulties regarding drug maintenance and permeation on the eye surface. Therefore, the in vivo fluorescence images of the ocular irritation test using eye drops were analyzed for the anti-inflammatory drug flurbiprofen (FB) and ophthalmic DDSs based on LDH-FB and LDH-FB coated with HA [211]. The results of release showed that even after 10 min of dropping, the retention time of the drug in the ocular surface was higher in the DDS form. The FB was still present after 30 min in the LDH-FB-HA indicating the promotion of the bioavailability by the composite delivery system.

Niclosamide (NIC), an antiviral drug that showed good efficacy combined with a vaccine in facing the virus SARS-CoV-2, has limited bioavailability and solubility. Thus, the use of a carrier can enhance its efficacy. NIC was intercalated into MgAl-LDH modified by the polymer Eudragit^®^ S100 (ES 100) and afterward coated by the Tween 60 (Nanoparticle, NP). The NP presented higher pharmacokinetic parameters (in vivo test by oral administration), maximum plasma concentration (C_max_) of about 15,000 ng/mL, the time required to reach the C_max_ (t_max_) was 0.60 h, and area under the plasma concentration-time curve (AUC) was about 14,000 ng.h/mL. These results are superior to those data observed for the commercial form Yomesan^®^: C_max_ of about 155 ng/mL, t_max_ of 4 h, and AUC of approximately 110 ng.h/mL. These results endorse the importance of using LDH to improve and combat diseases such as COVID-19 [212]. 

General disease treatments suffer from biodistribution drug effects which compromise health cells and organs [26]. An LDH dopped with copper and loaded with anticancer drugs 5-fluorouracil and albumin-bound paclitaxel was used to treat 4T1 breast cancer cells that are highly aggressive for lung metastasis [213]. The hematoxylin and eosin staining of the lung tissues showed that photothermal therapy of the composite material suppressed lung metastasis compared to free drug and chemotherapy. Hence, the use of LDH associated with photothermal therapy showed its ability to target cancer cells without compromising other tissues. In other work, the in vivo pharmacokinetic parameters of LDH stabilized by the poly((oligo(ethylene glycol) methyl ether acrylate)-block-poly(monoacryloxy ethyl phosphate) and having immobilized DOX (abbreviated as P-LDH-DOX) were evaluated after intravenous administration [214]. According to the study, the free DOX had a rapid terminal elimination phase (t_1/2β_ = 3.26 h) compared to LDH-DOX (t_1/2β_ = 24.02 h) and P-LDH-DOX (t_1/2β_ = 26.05 h). Therefore, the LDH promotes a longer circulation and retention time of the drug in the bloodstream, which improves the efficacy of the treatment.

LDH has been used to overcome the side effects commonly presented by drugs. The nanoparticle comprising LDH-albumin containing the antitumor drugs 5-FU and albumin-bound PTX (ABX) (abbreviated BLDH-5FU-ABX) was evaluated for colorectal cancer treatment, showing high efficacy in the induction of colon cancer cells apoptosis [215]. The in vivo anticancer efficacy analyzed by the tumor size of Balb/c female nude mice showed that the nanoparticle inhibited by 4 to 6 times the tumor growth compared to the BLDH-5FU or BLDH-ABX, which was even higher with the free drugs. Thus, the combination of 5-FU and ABX with BLDH increasingly improved cell apoptosis. The hematoxylin and eosin images did not show changes in the other organs (heart, lung, liver, spleen, and kidney), which demonstrated no overload by them after the application. Therefore, LDH can assist in cancer treatment, decreasing the side effects of the drugs. 

**Figure 15:**
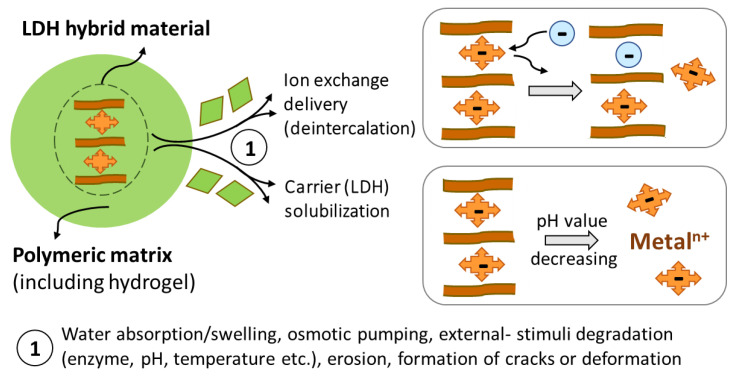
Mechanisms for the drug delivery from DDS based on polymer/LDH nanocomposites.

Considering the LDH properties and the data collected in the experiments mentioned above, the mechanism of drug delivery from DDS based on polymer/LDH nanocomposites occurs by the processes involving the polymer phase (water absorption/swelling, osmotic pumping, external-stimuli degradation, erosion etc.) and the LDH hybrid material (exchange reaction or carrier solubilization) (Figure 15). At the beginning of the release process, transformations should occur mainly in the polymer phase, moving on to a step in which both phases are changed. In view of the inorganic phase, deserves to be highlighted that the delivery rate should be affected by the solubility products of the metal hydroxides related to the LDH chemical composition, the material crystallinity (structural organization), the size and morphology of the particles, and the surface functionalization.

### 6.1. Polymer/LDH Nanocomposites for Oral Drug Delivery

Focusing on oral drug administration systems, nanocomposites display improved properties when compared to their components separately. In this regard, polymers used in biomedicine have desirable biocompatibility and biodegradability properties. However, they are often unstable in aqueous or saline solutions at different pH values, triggering a premature drug release, and frequently show low thermal stability [216]. On the other hand, although LDHs are widely explored as reservoirs of pharmacologically active species, they are highly soluble in acidic media, such as the stomach fluid (pH 1.2) [217], occurring dissolution and, consequently, the early release of the stabilized drug in the interlayer region, making it a drawback for oral administrations.

Thus, the combination of both organic and inorganic counterparts in one material becomes highly advantageous: the inorganic counterpart offers high surface area, low toxicity, enhancement of the therapeutic agent solubility, and thermal and chemical protection of the active compound [218]. On the other hand, besides providing higher biocompatibility with biological systems, the organic phase has the function of protecting the LDH-drug hybrid from drastic changes in pH, such as previous solubilization of the LDH fraction in an acidic environment (e.g., gastric juice) [219], contributing for decrease the possibility of first-pass effects, where the concentration of the intercalated drug in LDH may be significantly reduced, and the drug can be inactivated before reaching the systemic circulation. Therefore, the combination of polymers and LDH-drug hybrids to form nanocomposites support the controlled release of the active species owing to the decrease in both mobility of ionic drugs and their aggregation, besides overcoming drawbacks associated with traditional DDSs [220]. Hence, the preparation of LDH-based drug delivery nanocomposites is a strategy widely employed in the last two decades to preserve the release properties through the gastrointestinal tract [170,171].

With this strategy in mind, a core-shell nanocomposite was proposed by Li et al. [221], in which the LDH-fenbufen hybrid was the core, and the protective enteric polymers Eudragit^®^ S 100 or Eudragit^®^ L 100 acted as the shell. The presence of the carboxylate groups in the anionic co-polymers facilitates a strong interaction by hydrogen bonding between the polymer chains and surface hydroxyl groups from the LDH layers. In addition, in vitro experiments simulating the passage through the gastrointestinal tract demonstrated that the presence of enteric polymers as coating of the intercalation compound allowed a controlled release of fenbufen in an acid medium; the release was essentially completed after 3 h in pH 7.4 due to the dissolution of the polymer matrix at higher pH values.

From this seminal work to date, many studies with analogous purposes involving different polymers in LDH-based nanocomposites have been reported in the literature. For instance, xyloglucan, a natural polymer extracted from *Hymenaea courbaril* (jatobá, a native Brazilian plant) seeds, was employed at different concentrations (0.5 and 3.0% *w*/*v*) to protect the LDH-enalaprilate intercalation compound prepared by an ion exchange reaction [219]. The resulting nanocomposite, which was processed as powder, showed a heterogeneous core-shell structure with different nanometric sizes and distributions. In vitro studies of enalaprilate, released from the nanocomposites in a sequential experiment that simulate the different environments of the gastrointestinal tract, revealed that the system containing LDH-enalaprilate coated by xyloglucan at 3% (*w*/*v*) shows a slowed drug release (less than 60%), allowing the drug transit through the gastrointestinal tract. The associated mechanism involved the dissolution of the LDH counterpart and ion exchange between the intercalated drug and phosphate anions from the simulated intestinal medium.

On the other hand, the effect of the presence of LDH in a series of pH-sensitive nanocomposite beads prepared from the combination of LDH and alginate was explored by Zhang et al. [222]. The drug loading, swelling properties, and controlled release profile of diclofenac sodium adsorbed on LDH in the beads were investigated. The presence of LDH significantly increased the entrapment efficiency, modulated the swelling behavior according to the LDH content in the material, and improved the controlled release of diclofenac sodium from the nanocomposite beads. These effects were attributed to the electrostatic interaction between alginate and LDH, which probably restricted the polymer chains’ mobility, slowing down the swelling and dissolution rates of the beads in aqueous solutions.

Analogously, a pH-sensitive system for oral delivery of insulin from nanocomposite beads composed of alginate and LDH was also developed [223]. The strategy employed in this study was to combine a gel of alginate with ZnAl-LDH particles, and once formed the LDH-alginate hybrid hydrogel, the insulin was added to the medium, forming a single batch that was posteriorly processed as beads. The release of insulin from the nanocomposite beads was tested in the presence and absence of glucose phosphate in pH 7. The results demonstrate that from 60 units of loaded insulin, the LDH-alginate/insulin beads show 93% and 26% of insulin released in 70 and 50 min of the assay in the presence and absence of glucose phosphate, respectively. Interestingly, in a material prepared from the combination of LDH and insulin (in the absence of alginate) for comparison purposes, a release of 95% of insulin in 90 min of the test was noticed in the presence of glucose phosphate. However, in the absence of glucose, the releasing rate was about 50% of loaded insulin after 30 min of the test, where a reduction in the slope of the releasing plot was evidenced after this time. This behavior was attributed to a possible restoration of hydrogen bonding between the released insulin and hydroxide groups of LDH layers, causing a modification in the release of the peptide hormone.

Likewise, taking advantage of the positive charge of the LDH layers and the versatility of processing biopolymers as beads through ionic gelification, pH-sensitive nanocomposite hydrogel beads based on the combination of LDH-drug and CMC polysaccharide were proposed as a potential system for ibuprofen [224] and cephalexin [225] oral administration. Both drugs were previously intercalated into MgAl-LDH, forming the hybrid materials, and later incorporated in a gel of CMC. After homogenization, the resulting nanocomposite gel was stabilized as beads through crosslinking with Fe^3+^ ions from a FeCl_3_ solution. Considering that swelling studies can indicate the speed and easiness of a liquid diffusion into the nanocomposite bead, swelling properties of the prepared beads were carried out in buffer solutions at different pH values (1.2, 6.8 and 7.4). The results revealed a pH-dependent swelling behavior of the nanocomposite beads with increasing pH value; the ionization of carboxylic groups of CMC chains and the consequent increase of the electrostatic repulsion between the charged groups, and also the rise of osmotic pressure, promoted the materials’ swelling. The drug delivery profiles of both nanocomposite beads showed better protection against a stomach-like environment in comparison to LDH-ibuprofen or LDH-cephalexin alone while still allowing controlled release under the conditions of the intestinal tract. Such results indicated that these systems are very promising for achieving good control over the release of drugs that must pass through the gastrointestinal tract.

Similar results were also reported by Negati et al. [226], i.e., an increase in the concentration of CS in nanocomposite systems based on LDH-cetirizine decreased the release rate of the drug in pH 7, showing 50% of the drug released after 15 h of experiment with nanocomposite samples with 1 wt.% of polymer. The possible reason for observed sustained release behavior is that the interlayer region of LDH acts like a microvessel where the incorporated cetirizine species can be released via the ion exchange reaction, while the CS concentration can be adjusted to control the rate of cetirizine release from the LDH structure. Rezvani and Shahbani [227] studied the rate of drug release of core-shell systems based on ciprofloxacin (CFX) intercalated into ZnAl-LDH (core), protected by alginate or CS polysaccharides (shell) under pH conditions that simulate the sequence in the gastrointestinal tract (1.2 for 2 h, 6.8 for 2 h and 7.4 for 4 h). The results showed a lower release rate of CS/LDH-CFX (39% of cumulative drug release) in comparison to analogous materials prepared from alginate biopolymer, showing in the last case 96% of CFX release in the assay finalization. This behavior is mainly due to the leaching of Ca^2+^ ions employed in the crosslinking of alginate chains, increasing the solubility of the nanocomposite system in the simulated intestinal medium.

As explored throughout this review work, some synthetic polymers and currently more studied natural polymers are effective materials to compose the LDH-based formulations. This is the case of positively charged CS, which shows high sensitivity in acidic media (high swelling degree) and low sensitivity to neutral or alkaline environments (low swelling degree). The opposite behavior is observed using negatively charged polymers, such as alginate, CMC, xanthan gum or carrageenan, which are stable in acid conditions, but highly sensitive to basic media. To increase the resistance of such systems under conditions of high sensitivity and thus allow a better performance of the nanocomposite system in other areas of interest in the gastrointestinal tract, a strategy widely used is its combination with other polymers, in which they act as macromolecular crosslinking agents.

In this sense, polymeric blends are widely studied and associated with LDH as effective pharmaceutical carriers for the most diverse proposals [228]. Usually, the good interaction between the components, mainly driven by hydrogen bonds between polar groups of the polymers and hydroxide ions present in the LDH structure, results in a highly synergistic effect. Regarding this, Alcântara et al. [106] built up a nano-system of oral controlled delivery based on LDH-ibuprofen combined with alginate and zein biopolymers. The aim of this work was to improve the properties of the DDS using a hydrophobic protein combined with LDH-ibuprofen before the incorporation of the alginate matrix into the system. Besides the physicochemical characterization, the alginate-zein/LDH-ibuprofen nanocomposite, which was processed as beads, was evaluated for swelling properties and drug delivery in conditions that simulate the gastrointestinal tract. In vitro studies revealed that, due to the presence of the LDH together with the formation of a complex between the carboxylate groups of alginate, the amine groups of zein and hydroxide groups from the LDH fraction, the formulation was capable of delaying drug release, not only in acidic conditions but also in solutions that simulated intestinal fluid (pH 6.8 and 7.4, in which the alginate is very sensitive), allowing the drug release in a sustained manner in the medium, with preservation of the bead structure until the end of the test.

**Figure 16:**
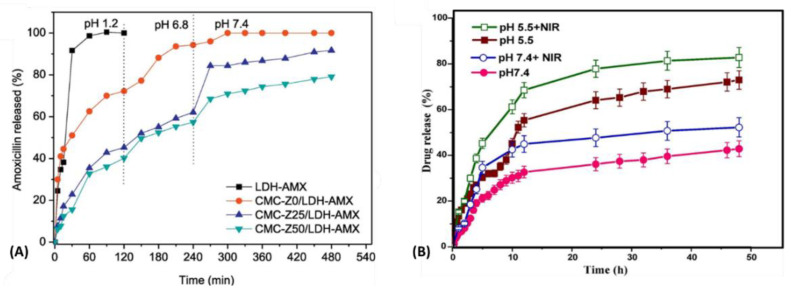
(**A**) Amoxicillin drug release profile at in vitro simulated gastrointestinal conditions (2 h at pH 1.2, 2 h at pH 6.8 and 4 h at pH 7.4, simulating the gastric juice and the first and second zone of intestinal fluid, respectively) from LDH-amoxicillin and CMC-zein coated LDH-amoxicillin systems (reprinted from Rebitski et al. [229] with permission of Elsevier) and (**B**) Cumulative DOX release profile at pH 5.5 and 7.4 in the absence and presence of NIR radiation from the LDH-based chemophotothermal nanocomposite system (reprinted from Anirudhan and Chithra-Sekhar [230] with permission of Elsevier).

In a recent work, the LDH-amoxicillin intercalation compound was also protected with a biopolymer blend composed of zein protein and CMC polysaccharide, giving rise to nanocomposite beads [229]. When compared to LDH-amoxicillin or pristine biopolymers blend, the nanocomposite beads, which were evaluated as a function of the amount of zein protein, demonstrated good compatibility between their components, providing a more controlled release of amoxicillin and enabling the active species to reach the intestinal tract, improving the drug’s bioavailability (Figure 16A).

Experiments following similar strategies to enhance specific properties of the carrier system and modulate the release of pharmaceuticals to a particular target region were reported. In this perspective, an effective oral controlled release system of 5-aminosalicylic acid (5-ASA) intercalated into LDH combined with CS and pectin was reported [231]. Considering that 5-ASA should act predominantly in the intestinal tissue due to its action in the treatment of intestinal inflammatory diseases (treatment of ulcerative colitis and Crohn’s disease, for instance), the LDH-5ASA was incorporated into a CS matrix with mucoadhesive properties and processed as beads coated with a thin layer of pectin, ensuring the stability of the CS at the acidic pH of the gastric fluid. The resulting nanocomposite proved to be a promising candidate for the targeted administration of 5-ASA because of its great stability in aqueous media, in addition to a controlled release of the drug over pH variations from the gastrointestinal tract, with a greater amount of drug dispensed in fluids that simulate the intestinal area, acting on the focus of the disease and minimizing possible side effects.

In an analogous approach, Abniki et al. [232] proposed a new formulation for the sustained delivery of mefenamate anions intercalated into MgAl-LDH for oral administration. To improve the loading and sustained release of the mefenamate drug, a co-intercalation of gum arabic together with the drug into LDH was investigated; subsequently, the resulting LDH-drug-gum arabic system was covered with CS biopolymer. A strong organic-inorganic interaction between the layers of LDH and gum arabic chains produced a slow release of the drug, while the presence of CS provided better control of the drug release in the intestinal medium.

Considering that the poor solubility of pharmacologically active species can be a limitation in drug delivery, it is essential to develop strategies that improve the solubility, which would result in better absorption of drugs, and consequently increase the drug’s bioavailability, as mentioned before. In this context, Pu et al. [233] developed nanocomposite beads based on MgAl-LDH and PLGA, a copolymer of PLA and polyglycolic acid, with the aim of increasing the solubility of danshensu, a component of *Salvia miltiorrhiza* (Danshen) applicable in cardiovascular diseases treatment. The strategy employed in the work was the previous exfoliation of LDH structure using formamide as solvent before the combination with danshensu extract. Then, the hybrid material was incorporated into PLGA suspension, which was processed as beads through the double emulsion solvent evaporation method. In addition to the enhancement of the drug solubility, physicochemical characterization indicated that the danshensu was successfully intercalated in LDH by an exfoliation reassembly process, reaching 32.6% of drug loading. The presence of LDH in the PLGA matrix contributed to reducing burst effects with a sustained release profile within 10 days. Moreover, the PLGA/LDH-danshesu nanocomposite beads were ineffective in the induction of hemolysis, suggesting that these materials show good blood compatibility and can be an effective DDS for oral administration.

In another study, multiple layers of pH-sensitive enteric copolymers (Eudragit^®^ copolymer S-100 and L-100) were coated over sulfasalazine-loaded LDH nanohybrids previously prepared using DMSO as a co-solvent [234]. This is a strategy commonly employed to improve the solubility of hydrophobic drugs and enhance the loading efficiency of the LDH-drug hybrids. The results demonstrated that the multiple layered enteric polymer coating preserved the LDH structure in the gastric environment, accelerated drug release efficiency and dissolution rate, along with increased oral bioavailability in the colon, likely due to emulsification properties of the Eudragit, showing sustained release that maintained the sulfasalazine concentrations in a therapeutic window. Furthermore, biological studies of the nanocomposite formulation through experiments employing superoxide radicals and lipid peroxidation suggested a highly protective effect against inflammation, while the histological view from the liver and kidney sections indicated that the Eudragit^®^/LDH-sulfasalazine nanocomposite is safe and highly biocompatible.

Taking into account that combination of immunization with antiviral medication formulation can be an effective technique for combating the current pandemic scenario, an oral formulation with Tween 60 and Eudragit S100^®^ that incorporate niclosamide-exfoliated LDH was proposed as the antiviral system against COVID-19 [212]. In vitro experiments showed that coating the particles with Tween 60 increased both the extent and rate of release compared to the uncoated sample under pH conditions of 1.2 and 6.8. Hence, a coating can be advantageous to improve niclosamide release, especially in the intestinal region. In comparison to the commercially available Yomesan^®^, the in vivo analysis revealed that the oral nanocomposite system with antiviral action was able to maintain a therapeutic relevant concentration of the drug in the plasma, demonstrating that the new formulation can be a successful oral DDS to treat SARS-CoV-2 viral infections. 

More recently, in order to obtain biocompatible magnetic systems of oral delivery of sodium diclofenac, Barkhordari and Alizadeh [235] developed nanocomposite beads based on LDH and CS that incorporate different amounts of Fe_3_O_4_ nanoparticles (0.05–0.15 wt.%). The release of sodium diclofenac adsorbed on the beads was investigated at different pHs and under the influence of an external magnetic field (EMF). Through the vibrating sample magnetometer as a function of the magnetic field at room temperature, the saturation magnetization for the DDS was 5360 emu/g, indicating that the coating of Fe_3_O_4_ by CS/LDH does not change the magnetization of the nanoparticles. This superparamagnetic property showed a significant effect on the sodium diclofenac release: the increasing amount of Fe_3_O_4_ nanoparticles in the nanocomposite beads becomes the release slower than to control samples, displaying a cumulative diclofenac release under EMF at pH = 1.2 and 7.4 and 9.3 in the range of 58.3–76.4% and 20.0–25.1 and 16.2-18.3%, respectively. These results indicated that the CS/LDH/Fe_3_O_4_/sodium diclofenac system has become more targeted and can be promising as magnetic pH-sensitive drug delivery.

Another reported strategy employed to obtain an innovative composite system of oral drug release endowed with magnetic properties described the incorporation of magnetic graphite nanoparticles into an alginate matrix containing ibuprofen between the layers of MgAl-LDH [231]. The analysis by different characterization techniques evidenced that the processed systems, such as films and beads, that contain magnetic graphite in their composition present a significant reduction in water absorption and an increase in the mechanical resistance of the wet material compared to the systems based on pristine alginate. Additionally, the in vitro kinetic results of ibuprofen release from the magnetic systems revealed a more sustained profile, wherein the release can be stimulated by an external magnetic field, allowing the modulation of the release levels and the delivery at a specific target site.

To increase biocompatibility and comply with essential requirements for promising oral drug administration, biocompatible LDH can also be used as a coating of nanoparticles. Since LDH has demonstrated an advantage in internalization across negatively charged biological membranes without the requirement for any extra post-modification or functionalization, this approach can be applied [236,237]. In this perspective, ZnAl-LDH, together with PVA and sorafenib antineoplastic drug, was used as co-coating of iron oxide nanoparticles by coprecipitation method in the preparation of a magnetic DDS [238]. According to reported data, the coating resulted in a narrower size distribution and decrease in particle size (around 40 nm), while the superparamagnetic nature of the iron oxide nanoparticles was preserved in the composite. Besides no cytotoxicity towards normal fibroblast cells, an enhancement of the antineoplastic effect of the magnetic system compared to free sorafenib against liver cancer and HepG2 cells was observed.

Considering this biocompatible property, numerous researchers have conjugated negatively charged cytotoxic drugs with LDH and successfully delivered them into the cells with features of controlled release [164]. However, scarce studies on LDH combined with polymers as oral devices for cancer treatment are reported up to now; most of them involve natural polymers in their composition. Pooresmaeil et al. [239] investigated the design of a new oral DDS for colon cancer treatment comprising 5-FU loaded in ZnAl-LDH (about 87%); the resulting hybrid was posteriorly encapsulated in a polymer matrix based on CMC. The presence of the biopolymer in the CMC/LDH-5FU material processed as beads induced a controlled and sustained drug release behavior, preventing the previous dissolution of the system in the simulated stomach medium; a high swelling and drug release in the alkaline medium were observed, indicating that these systems have pH-sensitive properties. The 3-(4,5-dimethyl-2-thiazolyl)-2,5-diphenyl-2H-tetrazolium (MTT) assay demonstrated the biocompatibility of the nanocomposite beads, yielding a potentially safe oral delivery system for colon cancer therapy.

Likewise, a new drug carrier based on MTX-loaded ZnAl-LDH and carboxymethyl starch (CMS) as an oral vehicle for specific colon tumors was recently developed by Pooresmaeil and Namazi [240]. The prepared DDS exhibited controlled and prolonged drug release in the gastrointestinal tract and high bioavailability. Additionally, CMS@LDH-MTX microspheres showed acceptable enzymatic breakdown and cancerous cell-killing ability, becoming a versatile therapeutic choice for the delivery of anticancer drugs to the intestinal area. Similarly, in another study, CMS was also used as a coating of simultaneously co-loaded 5-FU and DOX into MgAl-LDH to improve colon cancer therapy [241]. The results showed that coating the LDH-drug hybrids with CMS as a pH-sensitive biopolymer improved performance in cancer treatment exhibiting a controlled release profile in acidic conditions (about 22% and 29% of DOX and 5-Fu, respectively), allowing most of the loaded drug to be released into the intestinal environment. In addition, the cell viability tests against Caco-2 cells (colon cancer cell line) displayed that the nanocomposite material is biocompatible, likely due to the sustained release properties, which prevented the burst drug release of the anticancer agents.

Photothermal therapy (PTT) is widely used in physical cancer treatment, which is mediated by light. However, PTT alone can be a challenging treatment because of the uneven distribution of heat in the treatment volume [242]. Hence, integrating targeted chemotherapy with PTT would be a viable way to increase the effectiveness of cancer treatment. This approach was recently explored by Anirudhan and Chithra-Sekhar [230] in a study that describes the design and synthesis of a complex dual-sensitive nanocomposite platform based on the combination of LDH functionalized with isocyanate, covered with targeting ligand folic acid conjugated thiolated CS and gold nanoparticle (AuNp) for both targeted pH-responsive photothermal therapy against breast cancer using DOX as an anticancer molecule model. Thanks to interactions established between the components through electrostatic interactions, an improvement of encapsulation and loading efficiency of DOX was observed, showing a maximum of drug release at pH 5.5, which simulates the microtumoral environment, characterizing the system as pH-stimuli responsive (Figure 16B). In addition to the non-toxic nature of the system, quantitative flow cytometry analysis of the proposed formulation indicated a good targeting effect as well as increased destruction activity of cells than free DOX in MCF7 cells (breast cancer cells), while AuNp provided to the system better resistance to high temperatures by the near-infrared (NIR) light irradiation, enhancing the photothermal therapy and fluorescence imaging.

As aforementioned, many research groups around the world are working to develop more effective therapies with fewer side effects for millions of patients. Although LDH shows considerable potential for treating diseases like cancer, great challenges must be overcome in the biological environment, such as the (i) propensity to agglomeration in ion-rich surroundings, increasing its size and, consequently, promoting alterations in the colloidal stability, (ii) acidic degradation in the stomach and (iii) low bioavailability in the small intestine [243,244]. A possible approach to overcome such drawbacks is the modification of LDH with a sort of “camouflage," which can mimic other components of the biological environment. In this context, the interaction of LDH with polysaccharides or proteins (albumin, for instance) can give rise to changes in the physicochemical properties of the layered solid in a biological medium [48]. These possibilities of modifications, together with the excellent ability and good adjuvant function of LDH as a nanocarrier for protein antigen delivery, enhancing the immune response, yield potential constituents in oral vaccine delivery.

This was the strategy recently employed by Yu et al. [245] to obtain new nanocomposites based on LDH for enhanced oral vaccine delivery purposes. In this case, alginate-CS coated LDH@protein antigens (protein antigens = BSA, Albumin–fluorescein isothiocyanate conjugate (BSA-FITC)) have been developed, and the protein release properties were investigated at various pH values. For this, LDH@antigen was then coated with the CS, following a second polymer coating with alginate, using sodium tripolyphosphate (TPP) and CaCl_2_ as crosslink agents, respectively. The nanocomposite system displayed effective protection of the antigens and LDH from acidic solubilization. The flow cytometry analysis demonstrated that after the disintegration of the alginate layer from the oral vaccine device at the basic medium, the resulting CS-coated LDH could significantly improve the attachment and internalization of proteins at the Caco2 intestinal cells and macrophage, likely due to its strong interaction with the saccharide receptors and surface proteins. These studies demonstrated the high versatility and potential of LDH-based nanocomposites, opening new possibilities for the use of these materials as oral delivery systems for a wide variety of pathological conditions.

### 6.2. Polymer/LDH Nanocomposites for Transdermal Drug Delivery

The transdermal delivery of drugs from polymer/LDH nanocomposites can be achieved by two minimally invasive approaches, such as the use of gel formulations applied to the skin tissue [246] or through the formation of an array of microneedles [247]. Using the first approach, Kim et al. [246] developed a system based on a gel of Zn_2_Al-LDH intercalated with the model drug flurbiprofen (FB) and Eudragit^®^ S-100 at different concentrations. XRD data confirmed the intercalation of FB, and in vitro release study showed that Eudragit^®^ aided the release of FB from the layered material in comparison to the system without polymer. Possibly, Eudragit^®^ promotes the enlargement of the edges of the LDH particles, aiding the total out-diffusion of the drug after 24 h. The in vitro skin permeability test using mice model tissue revealed that the increasing polymer concentration fostered the FB release while the delivery system without polymer permeated at least two times less drug.

Yan et al. [247] employed a Mg_2_Al-chloride LDH phase to improve the mechanical properties of a CMC array of microneedles containing ovoalbumin (OVA) protein for transdermal delivery application using a silicon microneedle array male mold. In comparison to the traditional subcutaneous and intramuscular injections, this approach drastically reduces pain and needle stick injuries while it can be manufactured to target a specific layer of the skin. Upon application, the polymer phase is dissolved in the skin, and the delivery system releases the drug without yielding a sharp waste product. Elastic modulus and hardness of the hybrids at 5 wt.% of loaded LDH were significantly improved in comparison to pristine CMC, which could benefit the piercing process of the skin tissue. Additionally, the dissolution process of CMC after contact with the skin was not affected by the presence of the layered phase in human and pig skin. The in vivo comparison of the antibody production through subcutaneous injection and the microneedle showed a greater response after 14 and 38 days of the procedure in both pristine and CMC-LDH microneedles, confirming the potential of this novel transdermal delivery approach.

### 6.3. Polymer/LDH Nanocomposites for Ocular Drug Delivery 

Intercalation of bioactive compounds into LDH structure for drug delivery is an interesting strategy for the treatment of eye-related illness since topical administration of a drug is preferred in comparison to more invasive procedures, especially in the inner and posterior segments of the eye [248,249]. Several characteristics of these delivery systems are often required for effective therapeutic intervention, such as ocular bioavailability, long retention and minimal systemic exposure, while also improving the permeability across ocular tissues [250,251]. The formation of polymer/LDH-drug composites can also enhance several key characteristics of the final product: increase of permeation to the inner portion of the eye and rise of the retention time of the drug [166,252].

Xu et al. [251] studied the use of a composite based on a Mg_2_Al-LDH co-precipitated in the presence of glycylsarcosine-modified chitosan (GS-CS), pirenoxine sodium (PRN). CS exhibits good mucoadhesion and enhanced trans-cornea drug penetration, while glycylsarcosine can target the peptide transport-1 (PepT-1) protein to further enhance corneal permeation. PRN is widely used to inhibit the development of cataracts but exhibits low ocular bioavailability, which can be improved by intercalation in LDH matrices. XRD data revealed that the basal spacing of the GS-CS-PRN-LDH contains intercalated PRN, and it was further expanded when the GS-CS content was increased in the hybrids, indicating a combination of both intercalation and surface adsorption of the modified polymer over the LDH structure. The in vitro release study showed a fast initial release during 45 min, a slower release during the 45–300 min and a constant release until 360 min. This release profile is related to the initial release of PRN from the surface or near the surface of the LDH structure, and the slower release is associated with the ion-exchange reaction from the intercalated PRN. The increase of the GS-CTS content in the hybrid decreases the cumulative release of PRN owing to a slightly stronger host (GS-CTS-LDH)-guest (PRN) interaction. All analyzed GS-CTS-PRN-LDH systems exhibited a sustained release when compared to pristine PRN. Both GS-CTS-PRN-LDH and GS-CTS-LDH (PRN unloaded sample) showed a high degree of cell viability up to 75 μg mL^−1^ for human corneal epithelial primary cells (HCEpiC). Quantitative cellular uptake by HCEpiC showed that GS-CTS-PRN-LDH hybrids presented an uptake index over 3 times larger than pristine PRN and double of the PRN-LDH sample at 4 h of experiment.

The in vivo precorneal residence measurements of New Zealand albino rabbits showed that, in a commercial product, PRN could only be detected in the first 2 h after eye drop administration, while GS-CTS-PRN-LDH remained detectable for 6 h and the PRN concentration was significantly higher as well. The ex vivo fluorescence image of rabbit ocular tissue showed that CG-GS-FITC-LDH eye drops were strong in all ocular tissue, especially in the iris-ciliary and crystalline lens, and a higher content of GS-CTS in the hybrid material increased the fluorescence of the analyzed tissue, indicating an enhancement of the ocular bioavailability for PRN. Similar results were also observed for other GS-CTS-LDH eye drop systems intercalated with dexamethasone sodium phosphate (DEXP) for drug delivery to the posterior segment of the eye [253] or phacolysin for cataract treatment [250].

In the study developed by Sun et al. [254], LDH-based composite materials were employed for glaucoma treatment through a hydrogel/thermogel based on an amphiphilic copolymer, poly-(DL-lactic acid co-glycolic acid)–polyethylene glycol–poly-(DL-lactic acid co-glycolic acid) (PLGA-PEG-PLGA) containing the intercalated brimonidine (Bri) drug. After hydrothermal synthesis of the Mg_3_Al-Bri LDH phase, the inorganic content was dispersed on a solution containing the (PLGA-PEG-PLGA) polymer phase to produce the hydrogels. The in vitro drug release study showed that without the polymer phase (LDH-Bri), over 75% of the Bri content was released from the LDH in the first 15 min and up to 90% after 30 min of the experiment. On the other hand, the thermogel containing LDH-Bri (LDH-Bri-thermogel) sustained the release of over 75% of the Bri content after 2 days and with a significant reduction in the initial burst release. This effect can be attributed to the polymer that causes a significant decrease in the ion exchange reaction of Bri from the LDH structure and/or slower diffusion of Bri from the thermogel. Cytotoxic evaluation using human corneal epithelial (HCET) cells showed that none of the components of the DDS decreased cell viability. The drug concentration in the aqueous humor of New Zealand rabbits was initially smaller for LDH-Bri-thermogel in comparison to the Alphagan^®^ commercial eye drops during the first 3 h but remained close to three times more concentrated after 24 h. After 48 h, the detection of Bri from the Alphagan^®^ group was almost zero, while it was still detected from the LDH-Bri-thermogel system after 7 days. The hybrid formulation sharply decreased the intraocular pressure in the first 3 h of the experiment and kept it consistently lower than the Alphagan^®^ group at 24 and 48 h.

## 7. Organic Polymers and Layered Double Hydroxides Nanocomposites for Tissue Engineering

Tissue engineering comprises the preparation of scaffolds to replace, repair, or reconstruct part or entire tissue (bone, cartilage, skin, blood vessels, muscle etc.) and is classified into two types: skin tissue engineering and bone tissue engineering [255].

### 7.1. Polymer Composites and Skin Tissue Engineering

Wounds consist of lesions coming from local trauma or originating from pathological conditions. Although the wound healing process presents particularities according to the tissue and local affected, the skin wound healing process illustrates the repair steps for the majority of tissues [256]. Since polymer/LDH composites application for skin wound healing is more studied in relation to other tissues, hereafter, the process of skin repair will be provided. Skin wound healing can be illustrated as a sequence of four linear steps, which may overlap over time: (1) homeostasis, (2) inflammation, (3) proliferation, and (4) remodeling step [256,257]. It involves different types of cells, such as keratinocytes, fibroblasts, immune, endothelial, and progenitor cells. These events are controlled by growth factors, chemokines, and cytokines. Metallic ions are also involved in the wound-healing process due to their implications for cell behavior and enzymatic activity regulation [258]. The action of these and many other chemical identities are coordinated by a signaling network formed by a family of interleukin proteins that regulate the immune response and growth factors, coordinating cell-cell and cell-ECM interactions necessary to skin healing [257].

In the first step of wound healing (homeostasis and coagulation), platelets circulating into the blood stick to the damaged tissue keeping the blood in and protecting the body from microorganisms and dirt. Mediators that cause localized vasoconstriction are released, and the protective barrier of the skin is restored. Then, coagulation factors are activated, which will direct the deposition of fibrin proteins. Platelets and fibrils form a clot. At this point, homeostasis is restored. The clot mitigates hemorrhage and serves as a preliminary matrix for cell migration by releasing scaffold proteins such as fibronectin, vitronectin, and thrombospondins, allowing for the migration of keratinocytes (primary types of cells in the epidermis), immune cells, and fibroblasts. This step takes from minutes to hours.

Zinc cations play a role in keratinocytes differentiation and function, thus being an important metal in the first step of wound healing. In fact, Zn^2+^ dietary or inherent deficiency is associated with skin diseases, such as alopecia and dermatitis [259]. Platelet degranulation also leads to the release of inflammatory mediators and activates the complement cascade [260]. In the second step, which overlaps with homeostasis and occurs in the first 72 h after wound occurrence, histamine released by the activated complement cascade causes capillary dilation, accelerating the migration of inflammatory cells into the wound area. Pro-inflammatory signals make neutrophils and monocytes migrate and infiltrate the wound region, eliminating pathogens, dead cells, necrotic tissue, foreign debris, and toxic metabolites. Neutrophils phagocyte pathogens kill them through the release of ROS, proteases, or antimicrobial proteins.

The death of injured cells and the consequent release of cellular content are understood by the body as a danger signal that is recognized by tissue-resident cells, making them express several genes coding for chemical mediators responsible for propagating the inflammatory response. After fulfilling their function at the injured tissue, neutrophils undergo apoptosis and are uptake by macrophages triggering the system out of the inflammatory phase. At this point, macrophages, which transited from an inflammatory to an anti-inflammatory state, produce Vascular Endothelial Growth Factor (VEGF), promoting vessel growth and vascular anastomosis, in the called angiogenesis process, which re-establish vascular channels. Concomitantly to the angiogenesis, granulation tissue (which is composed primarily of type-III collagen (COL-III), fibroblasts, and new blood vessels) is also formed. From days to several weeks after wound occurrence, the fourth and last step takes place. In the remodeling and last step, angiogenesis slows down, and type-I collagen (COL-I) replaces COL-III making tissues stronger. This step starts two to three weeks after skin injury and can last from months to years [256]. It consists of the synthesis and reorganization of the EMC.

Wounds can be classified as acute or chronic. Acute wounds reach the complete cure, usually take no more than 12 weeks to heal, and present fewer chances to originate prominent scars. Chronic wounds, on the other hand, tend to recur, to persist for more than 12 weeks, and scars are more expected [261]. Impaired tissue regeneration is aggravated by age and diseases such as diabetes mellitus, ischemia, and hypertension [262]. Chronic wounds affect millions of people, and elderly persons (65 years and older) account for 85% of the cases [262]. In just three decades, the number of older adults is expected to more than double, driven by Eastern and South-Eastern Asia and North and sub-Saharan Africa [263]. The replacement of damaged tissues (including skin, bones, and vascular systems) follows the constant increase in global life expectancy, and that non-healing wounds result in enormous health and financial burden to individuals and the health care system, leading to morbidity or even mortality. Hence, discovering technologies able to assist tissue regeneration is of great interest. 

In fact, the global chronic wound care market, valued at 11.61 billion in 2021, is projected to grow to $19.52 billion by 2029 [264]. Although the increasing demand for treatments is apparent, solutions for acute delayed wounds and chronic wounds remain challenging.

Delayed acute wounds and chronic wounds frequently enter a state of pathologic inflammation due to a postponed, incomplete, or uncoordinated healing process. In these cases, the inflammatory phase of the healing process cannot be resolved and then is prolonged, affecting angiogenesis and ECM deposition. These scenarios can also be due to or intensified by bacterial infections [257]. Polymer composites as implantable devices or wound dressings for assisting wound healing may act in preventing infections, improving specific cells’ attachment, migration, and differentiation, or performing a targeted or local release of bioactive BL that can assist the wound healing process in one or more phases of wound healing. Regarding wound dressings, they were originally designed to cover the wound, providing a beneficial microenvironment for successful healing by controlling wound moisture and absorbing excess exudate [265]. Although the urgency in the development of solutions to treat severe or chronic wounds is evident, only four therapies were approved by FDA. Three of them consist of dermal substitutes or equivalent, while one therapy involves recombinant human platelet-derived growth factor [257]. No therapy with small-molecule drugs has been approved up to now [174]. Some challenges to the development of products for non-healing chronic wounds include scarcity of biological models (chronic wounds are uncommon in animals and difficult to simulate) and challenges in clinical trial execution and in drug delivery (release kinetics modulation) [266].

Effective wound dressings are expected to interact actively at the molecular level with biological structures guiding the wound-healing process. In more detail, they can locally alter the wound’s environment by targeting bacterial load and excessive protease levels, providing exogenous COL or species that stimulate or participate in COL neogenesis, and can also provide drugs responsible for reducing local pain. In general, wound dressings consist of natural or synthetic polymers able to be processed by many diverse methods, as previously discussed. Natural polymers, such as CS, gelatin, COL, and HA, have been studied due to their bioactivity and hydrophilicity, which are interesting to abstract exudate. Many synthetic polymers employed in tissue regeneration, such as PCL, PGA, and PLGA, present high hydrophobicity, which hinders the control of wound moisture and the interaction with tissues and body systems. Moreover, they are, in general, biologically inert. On the other hand, the synthesis of polymers provides versatility in composition and structure, thus allowing the tuning of the polymer properties [265]. The employment of LDH for the preparation of biomaterials for tissue regeneration offers noticeable advantages. The interlayer space of the LDH structure can accommodate several types of bioactive payloads of interest for the design of active wound dressings, like antibiotics, anti-inflammatories, and statins. LDH layer solubilization can provide metallic cations (such as Mg^2+^ or Zn^2+^) playing a role in different phases of wound healing, acting in the metabolism and/or in the activity of key cells, and/or having antimicrobial properties.

The incorporation of LDH particles in the polymeric matrix may surpass the limitations of both LDH and polymer. On the one hand, the surface morphology and porosity of the polymeric matrix assist cells adhesion, migration, and proliferation; the polymer supports and immobilize LDH solid particles, offering an additional barrier and possibilities to modulate the release of intercalated species and the degradability rate to follow the wound healing progression. On the other hand, LDH increases the hydrophilicity, improves the biocompatibility of the polymer, and allows the loading of hydrophilic bioactive species incompatible with hydrophobic polymers. LDH can also enhance the mechanical performance of the polymeric matrix. Thus, LDH/polymer composites are strong candidates for the modern generation of wound dressings.

**Figure 17:**
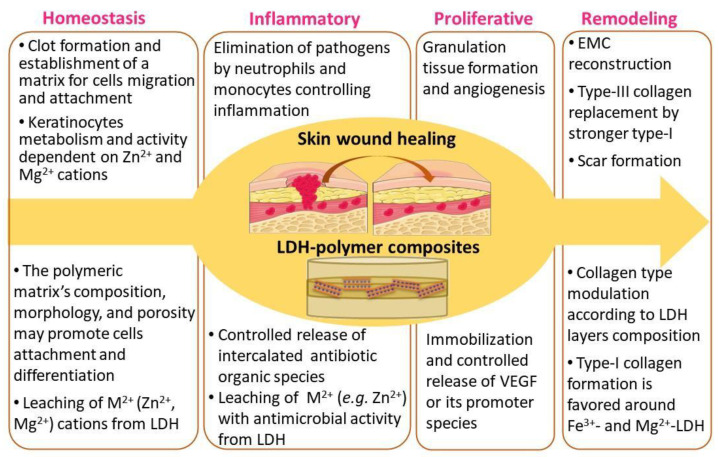
Progression of wound healing: some characteristics of each step of wound healing (top description) and the corresponding possible contribution of polymeric matrices and LDH (bottom description).

Concerning specifically the role of LDH in tissue healing, the repair of intramuscular tissues of mice around LDH tablets through the deposition of different COL types, according to the metal cations in the layers, was observed. For instance, COL-I and COL-III are predominant when Mg_2_Al-Cl and Zn_2_Al-Cl LDH are implanted, respectively [142]. Interestingly, when the same experiment was performed with LDH where half of the Al^3+^ cations were substituted by Fe^3+^ (Mg_2_Fe_0.5_Al_0.5_ and Zn_2_Fe_0.5_Al_0.5_), COL-I was predominant, prevailing over the divalent cation action [143]. The substitution of exogenous Al^3+^ cations by endogenous Fe^3+^ is of great interest. However, there is a concern about the possibility of iron excess causing oxidative stress and overpopulating the wound region with macrophages, which could impair wound healing [267]. Undoubtedly, the possible negative effects of iron are dependent on its dose. Furthermore, Mg_2_Fe_0.5_Al_0.5_ and Zn_2_Fe_0.5_Al_0.5_ LDH were intercalated with anti-inflammatory naproxen (NAP) and submitted to drug release assay in simulated medium to quantify not only NAP but the metals by ICP-AES technique [143]. Differently from Mg^2+^ and Zn^2+^, no signal for Fe^3+^ cation could be obtained due to its very low solubility product. Thus, a very low amount of free Fe^3+^ cations was leached from LDH, already able to play a role in COL neogenesis but not high enough for possible deleterious effects. In fact, the Fe^3+^ LDH samples presented excellent in vivo biocompatibility. The higher presence of macrophages in comparison to the studies for Al^3+^-LDH, but in the absence of other inflammatory cells, suggested a possible influence of iron in macrophage mobilization. Figure 17 brings aspects of each step of wound healing in which the polymeric matrix and LDH may act to assist the process.

**Figure 18:**
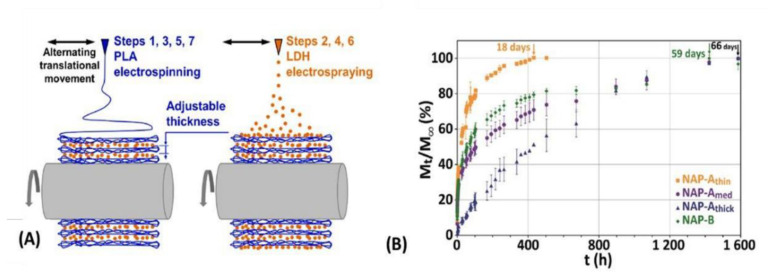
(**A**) Preparation of the composite membranes and (**B**) NAP anions release percentage as a function of time for the different composites (reprinted from Figueiredo et al. [73] with permission of Elsevier).

Some works showing different polymer/LDH composites aimed at modern wound dressings are commented ahead. Looking for a membrane design for an effective release of bioactive payload in the wound site, Figueiredo et al. [73] designed sandwiched membranes prepared by alternating electrospun PLA and electrospray LDH layers (Figure 18A). The Mg_2_Fe_0.5_Al_0.5_ LDH intercalated with NAP anions (Mg_2_Fe_0.5_Al_0.5_-NAP) were employed as model structures. Changing only the thickness of the electrospun polymeric layers, NAP anions release persisted from weeks to months. Such an approach can be employed for different systems, and the drug release is tuned according to the drug’s bioactivity.

Mg_2_Fe_0.5_Al_0.5_-NAP and Zn_2_Fe_0.5_Al_0.5_-NAP LDH particles were also employed by Figueiredo et al. [65] for the development of casting membranes based on the polyether-block-polyamide block copolymer (PEBA) aiming wound dressings preparation. LDH particles improved the mechanical properties of PEBA. Interestingly, by changing the divalent cation in the LDH layers, faster (for Mg-based LDH) or slower (for the Zn-based LDH) drug release was observed. LDH particle immobilization prevented normal human dermal fibroblasts from particle overload and exacerbated cytotoxicity. Moreover, LDH-NAP/PEBA membrane presented higher cell viability in comparison to the membrane containing non-intercalated NAP anions or containing the pristine Mg_2_Fe_0.5_Al_0.5_-Cl particles.

Mohammadi et al. [268] developed a wound dressing consisting of a polyurethane elastomer containing Mg_3_Al particles intercalated with an antibacterial curcumin macrocyclic calixarene complex. The polyurethane elastomer was synthesized from ricinoleic acid, also called castor oil, a polymeric raw material containing reactive-OH groups. Curcumin, a natural product, presents antibacterial activity and accelerates skin wound healing, acting in early and advanced steps of the healing process. In that investigation, curcumin was pre-immobilized in the macrocyclic calixarene complex and then the complex into LDH, aiming to surpass low chemical stability, rapid metabolization, and weak bioavailability. The composite containing 1 wt.% of LDH, in addition to the control composites consisting of LDH-NO_3_ and LDH, intercalated only with curcumin, was prepared by in situ polymerization. The composites containing Mg_2_Al-NO_3_ LDH presented antibacterial activity against *E. coli*, attributed to the high surface area/volume ratio of LDH nanosheets and their positive electric charge. The composite containing LDH-curcumin complex presented antibacterial activity for both *E. coli* and *S. aureus* strains, including accelerated wound healing.

**Figure 19:**
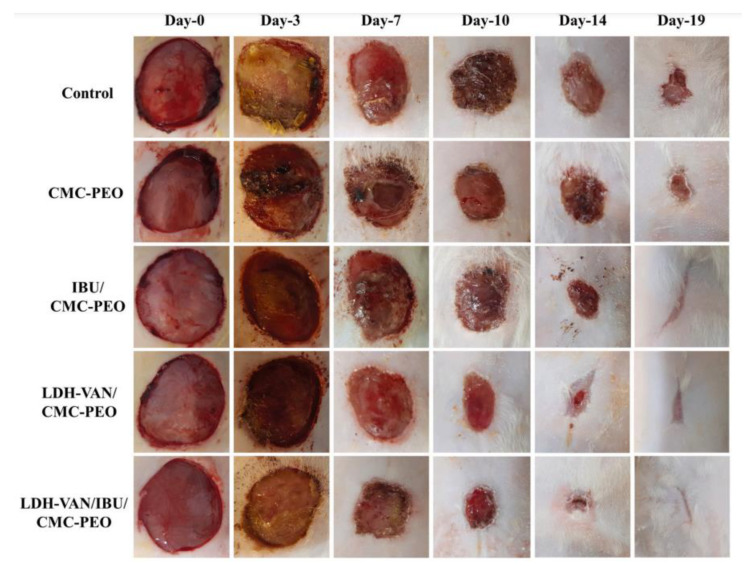
Representative photographs of wounds treated by control, CMC-PEO, and IBU/CMC-PEO, LDH-VAN/CMC-PEO, and LDH-VAN/IBU/CMC-PEO groups on days 0, 3, 7, 10, 14, and 19. (reprinted from Yoosefi et al. [269] with permission of Elsevier).

Yoosefi et al. [269] designed CMC-PEO bionanocomposite electrospun fibrous mats for wound healing containing ibuprofen (IBU) drug and Mg_2_Al LDH intercalated with vancomycin (LDH-VAN) for simultaneous delivery of analgesic and antibiotic species, respectively. The tensile strength of the fibrous mats was enhanced in the presence of LDH particles in comparison to the control (CMC-PEO mat). Figure 19 shows the visual aspect of the wounds in rats treated with mats. The following crescent order of wound healing improvement was observed: control < CMC-PEO < IBU/CMC-PEO < LDH-VAN/CMC-PEO < LDH-VAN/IBU/CMC-PEO.

### 7.2. Polymer Composites and Bone Tissue Engineering

Procedures for bone replacement or repair have been consistently investigated over the past years due to an increasing demand caused by different factors such as the growing older population, fractures, and bone defects and diseases [270,271,272]. Bone is not a static or isolated organ but multifunctional, metabolically active and integrated with other body organs [273]. The bone structure is constantly renewing itself during life, undergoing a remarkable rotation compared to other types of tissues present in the body. The processes of bone formation and resorption must be precisely regulated, guaranteeing their integrity to avoid bone fragility and, consequently, its injury. Bone regeneration is observed in the normal healing of a fracture in human and veterinary orthopedics and in more complex processes such as bone defects caused by trauma, infection, removal of tumors, or osteonecrosis (a disease caused by lack of blood supply to the bones), atrophic non-union (the bone starts to disappear because it is resorbed) and osteoporosis.

Several strategies are used to increase the process of bone regeneration in the dental and medical areas, such as autologous bone graft (i.e., the bone comes from the same individual), allograft implantation (i.e., material obtained from an individual of the same species), use of osteoconductive scaffolds (that is, which allow the juxtaposition of new bone tissue on its surface), or osteogenic distraction (a form of tissue engineering) [274]. The third generation of biomaterials for bone regeneration involves biodegradable materials to allow bioresorbability during the formation of new healthy bone and usually porous scaffolds instead of monolithic or bulk phases [275].

The scaffold for bone regeneration can be of different chemical nature: metallic (or metallic alloys), ceramic or polymeric. However, composites have properties more suitable for use as scaffolds. The human bone itself is an inorganic-organic composite, made up of approximately 70% of the inorganic phase (containing mainly calcium, phosphate, and carbonate ions) and 30% of the organic phase, with a predominance of the COL polymer [274]. Thus, tissue repair in orthopedics or dentistry has aimed at the use of biomimetic materials, that is, composites formed by phases that have properties close to those of bone in terms of structure, mechanical properties, bioactivity and osteoconductivity, to achieve the required therapeutic efficiency. Furthermore, scaffolds can host bioactive agents (growth factors, small peptides, drugs) released into the biological environment in a controlled way. Tissue engineering is based on a tripod consisting of scaffolds, biochemical agents, and cells.

The use of poly(methyl methacrylate) (PMMA) cements for orthopedic procedures became the gold standard because of their mechanical properties and safe characteristics for such purposes [270,276]. PMMA is biologically inert but not biodegradable, hindering bone growth over the polymer structure [272]. Furthermore, PMMA polymerization is an exothermic process that can cause tissue necrosis [276], requiring the incorporation of heat-stable antibiotics to address possible infections [270]. If the polymer used as support for cell adhesion and proliferation is biodegradable, chemical or enzymatic reactions lead to its disappearance until the end of bone repair. Biopolymers such as COL, CS and alginate are preferred over synthetic ones because they have comparatively high performance and low environmental impact, as they are abundant, renewable, inexpensive, and have low molecular weight. A new generation of scaffolds also includes nanoparticles (fillers) that, if they are also degradable, produce the known *green composites*.

These limitations can be minimized, and even novel properties on polymer-based materials can be achieved, by the presence of a wide variety of inorganic structures. Especially for bone-related applications, hybrid materials based on the combination of LDH and polymers are promising to owe to: (i) the enhancement of the mechanical properties of the resulting composite [271,277]; (ii) the improvement of osteogenic differentiation associated with the release of Mg^2+^ ions from the LDH structure and the alkaline microenvironment [272,278]; (iii) good biocompatibility, low toxicity and structural homogeneity [169]; (iv) thermal stability and drug release of bioactive compounds from the intercalated LDH structure [279]; (v) thermal insulation from the exothermic polymerization process, and (vi) creation of surface irregularities that could benefit osteointegration [272].

**Figure 20:**
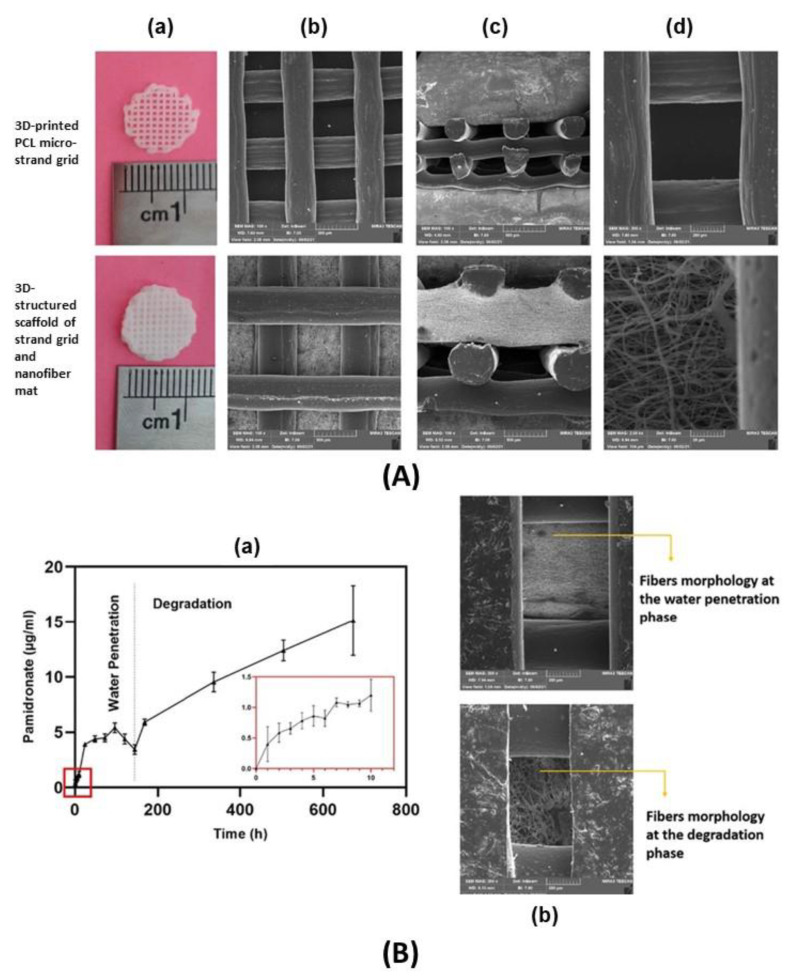
(**A**) Structural characterization of the PCL-based scaffolds: (**a**) macroscopic images; (**b**) top view (scale bar: 500 μm), (**c**) cross-sectional view (scale bar: 500 μm), and (**d**) top view of a pore of a 3D-printed grid (scale bar: 200 μm), and electrospun nanofibers (scale bar: 20 μm); (**B**) In vitro drug release: (**a**) cumulative release of Pam and (**b**) images of PCL/LDH-Pam fibers in the scaffold at the water penetration and degradation phases (scale bar: 200 μm), after 28 days of immersing in PBS solution (reprinted from Belgheisi et al. [280] with permission of Elsevier).

An example of these scaffolds, developed by Belgheisi et al. [280], is shown in Figure 20A. A 3D structure of PCL micro-strands grid was combined with nanofibers of PCL containing dispersed particles of pamidronate (Pam) drug intercalated into LDH (LDH-Pam) obtained by electrospinning (PCL/LDH-Pam). The delivery behavior of the drug in the PCL/LDH-Pam fibers glued in the 3D PCL grids, followed by 28 days in PBS medium, showed two stages of the controlled release: water penetration (firstly 7 days) and degradation (Figure 20B). The first stage occurs by ion-exchange sustained release event. After 28 days, the surface degradation by the PCL ester bonds hydrolysis began, exposing more LDH-Pam hybrids and keeping the gradual release of the drug. The data about activity of alkaline phosphatase (ALP), which indicates the osteogenic effect, showed a better behavior of PCL/LDH-Pam compared to PCL-scaffold without LDH-Pam in the MG-63 cells. Furthermore, changing the divalent cation in the LDH structure, a higher osteoconductivity was noticed for Mg-LDH than for the Ca-LDH. Therefore, the PCL/LDH-Pam composite shows potential for local drug delivery application in bone tissue engineering.

A study conducted by Wang et al. [272] investigated the formation of composites based on PMMA, Mg_2_Al LDH and mineralized COL-I. A mixture of methyl methacrylate (MMA) monomer, pre-polymerized PMMA and LDH and/or COL-I, submitted to polymerization reaction at 25 °C, produced a composite placed into molds to generate samples with different shapes. The presence of both COL-I and/or LDH in PMMA did not change the curing time of the polymer matrix. In vitro differentiation assays of human bone marrow mesenchymal stem cells (hBMSCs) showed a larger presence of the ALP-positive cells in PMMA-LDH and PMMA-COL-I-LDH than in pristine PMMA or PMMA-COL-I samples. Apparently, more calcium nodules were present in the ECM of hBMSCs cultured over the surface of modified PMMA materials in comparison to the pristine polymer, demonstrating the osteogenic capability of PMMA-LDH hybrids. When analyzing the skull bone growth into the PMMA-based materials using New Zealand white rabbits, it was observed that the samples containing LDH grew bone volume over 17 times higher than pristine PMMA. Tissue analysis (heart, liver, spleen, kidney, and spinal cord) of the rabbits showed no evidence of these structures exhibiting in vivo biocompatibility of COL-I and/or LDH-modified PMMA hybrid materials.

Furthermore, the use of new polymer-based hybrid materials was proposed as a promising candidate to replace PMMA-based cements. In one of these studies, Enderami et al. [271] evaluated the osteogenic differentiation of bone cells using highly porous PCL scaffolds containing a Mg_2_Al LDH intercalated with chloride ions to overcome the poor bone cell adhesion over the hydrophobic surface of the pristine polyester. This polymer has a low melting point (60 °C), is biodegradable and can be processed through different techniques for bone implant applications, such as electrospinning, 3D-printing, salt leaching and also thermally induced phase separation (TIPS) [281]. TIPS can promote the formation of a porous structure with a wide range of pore sizes, which can assist bone integration into the scaffold. To produce the PCL-LDH composites by TIPS, different concentrations of LDH-Cl (0.1, 1.0, and 10 wt.%) were dispersed in 1,4-dioxan and later mixed with a solution of PCL dissolved in the same solvent, frozen at −72 °C and then freeze dried to remove the dioxane. Regarding the mechanical properties, the compressive modulus of the composite was up to twice the value observed for pristine PCL at a moderate concentration of LDH, while the maximum displacement decreased owing to the presence of the inorganic particles. The porosity of the samples was not affected by the presence of LDH particles and remained high (94–96% range). The results of in vitro degradation indicated a faster rate when increasing the LDH content in comparison to the pristine PCL, which can take up to 24 months to fully degrade, which took less than 3 days for the 10 wt.% PCL/LDH sample. The results from in vitro bioactivity revealed the presence of apatite layers in all samples, but the number of mineralized spots was higher in the composite materials in comparison to the pristine PCL. Cell viability assessment revealed that the cells attached to the surface of composite scaffolds grew rapidly, indicating the presence of a cell-friendly microenvironment, while cell attachment investigation using hBMSCs indicated a regular distribution of the stem cells on all porous samples. The assessment of osteogenic differentiation of the hBMSCs showed that ALP and Runt-related transcription factor 2 (RUNX2) expression patterns were significantly higher for the 1.0 wt.% PCL/LDH in comparison to pristine PCL after 14 days, reinforcing the role of LDH particles on the differentiation process of stem cells to generate bone structure.

Cámara-Torres et al. [270] investigated the use of poly(ethylene oxide terephthalate)/poly(butylene terephthalate) (PEOT/PBT) block copolymers containing 5, 10 and 20 wt.% of Mg_3_Al intercalated with CFX to promote tissue regeneration and prevent bone infection. These composites were prepared using a twin-screw extruder by mixing PEOT/PBT pellets and the previously synthesized Mg_3_Al-CFX. Then, the scaffolds were produced from the pelletized hybrid material by melt extrusion, yielding porous cylindrical structures composed of stacked submillimetric filaments with pore size larger than 300 µm (overall porosity around 50%). Surface analysis of the scaffolds indicated a good integration of the LDH structure over the polymer matrix; the surface roughness was comparable to those of pristine PEOT/PBT scaffolds. The in vitro release of CFX from the polymer/LDH scaffolds indicated a sustained release over 1 month and a direct correlation between the amount of antibiotic delivered and the LDH-CFX loading. Since less than 20 wt.% of CFX was released from the polymer/LDH scaffolds at the highest loading tested and the cumulative release plateau was not reached during the evaluated period, it was proposed that only the particles located at the surface of the filaments were able to release the antibiotics. Even though CFX thermal decomposition occurred to some extent by extrusion technique, the scaffolds preserved antimicrobial activity against *P. aeruginosa* and *S. epidermidis* during the 1-month evaluation. The cytotoxicity of the composite investigated using human mesenchymal stromal cells (hMSCs) indicated no difference in cell viability regardless of the concentration of the LDH-CFX employed once it eliminated the initial burst release of antibiotics from the surface of the scaffolds. Calcium deposits were observed only after 28 days of experiments for the different samples tested and were absent on the highest LDH-CFX scaffold loading even after 49 days, indicating that CFX exhibits a tendency to hinder the mineralization step.

LDH-based hybrid materials were also proposed for bone-related applications when combined with pristine or modified natural polymers to improve their physical and biological properties. Alarçir et al. [278] combined a Mg_4_Al intercalated with carbonate ions, gelatin methacryloyl (GelMA) and alginate to produce printable bioinks for additive manufacture to generate biocompatible hydrogel scaffolds. The presence of the inorganic phase in the composite aided the filament formation and the stackability of layers of filaments to yield different printed shapes. In these composites, the presence of LDH also resulted in lower in vitro degradation rates in all evaluated loadings in comparison to the samples without LDH since the GelMA/alginate blend fully degraded after 4 weeks of the tests. In vitro cell adhesion studies also revealed that osteoblasts were attached to the surface and spread over the scaffolds in all LDH loadings during the 7-day evaluation. The osteoblasts cells were also added to two different bioink compositions (one containing 3.0 wt.% of LDH to the GelMA content and another without LDH) to assess their survivability upon the bioprinting process to produce the hydrogels to assist bone growth. The results showed that the scaffold containing LDH generated over 20% larger cell area and osteoblasts with a higher aspect ratio than the bioink without LDH, resulting in a larger cell spreading area.

Chen et al. [282] investigated the use of a Mg_3_Al LDH intercalated with carbonate ions and CS at high inorganic loading (33 wt.% of LDH to the CS mass) to produce porous structures by the freeze drying process, loading pifithrin-α (PFTα) after the contact with different concentrations of its solutions (10, 20 and 40 µmol L^−1^). PFTα is a selective inhibitor of the p53 protein associated with the hindering of the bone growth process. The CS/LDH scaffolds exhibited 3D interconnected macropores with pore sizes around 100 µm owing to the removal of the ice from the frozen structure upon the freeze-drying process; inorganic platelets were detected over the surface and in the inner parts of the scaffolds. XRD data suggested that LDH maintained its structural integrity even after contact with the acetic acid solution required to solubilize CS. All concentrations showed a fast release rate of the inhibitor in the first 15 h and then gradually reached a release equilibrium and did not exhibit significant differences in their drug release properties. However, osteogenic differentiation of hBMSCs was increased when PTFα concentration on the scaffold was high, which was related to a decrease in the protein level of p53 in hBMSCs, according to the western blot data. All CS/LDH-based scaffolds tested (with and without PFTα) exhibited similar adhesion and proliferation of hBMSCs, showing good biocompatibility of all components. In vivo experiments using Sprague-Dawley rats and implants of CS/LDH and CS/LDH-PFTα (40 µmol L^−1^) showed a higher amount of new bone formation, bone mineral density and total bone volume/tissue volume in the cranial defects treated with CS/LDH-PFTα after 12 weeks of the procedure. Hence, the CS/LDH nanocomposite loaded with an osteogenic molecule showed properties expected for local DDS in bone regeneration (Figure 14).

## 8. Conclusions

Several polymers and inorganic materials, such as layered double hydroxides, have been studied for decades and applied for the development of pharmaceutical formulations and medical devices, showing the basic requirements for proper contact with the human body, such as biocompatibility and metabolization. Additionally, each phase owns multiple and interesting properties allowing the development of original treatments, through different administration routes, for several diseases and assisting injured tissues and organs to heal. For instance, both LDH and polymeric matrices can intercalate and load bioactive species, respectively, and be superficially and structurally modified to achieve specific morphologies and superficial reactivity. Several metal cations able to form the LDH structure play a role in tissue regeneration. Moreover, changing the composition of the layers (i.e., M^2+^ and M^3+^ metals and the M^2+^/M^3+^ molar ratio), the particles morphology, and the degree of particles aggregation (according to the synthesis method, samples aging, and dispersive treatments), it is possible to adjust the metals leaching and the drug release kinetics. The chemical nature of the polymers dictates hydrophilic/hydrophobic balance, degradation kinetics, and physicochemical properties. Additionally, polymers can be processed through several techniques, which allow the production of 2D and 3D objects, porous and fibrous structures, with tunable physical properties (i.e., elasticity, toughness, density). Therefore, the combination of LDH and polymers, intimacy-related in the form of composites, offers a great opportunity to assist body functions and metabolism according to their complex and clever way of working.

More specifically, the association among drugs, LDH, and polymers have shown great enhancement in bypassing some usual problems faced by a DDS when interacting with the cells and the bloodstream. The factors impairing drug-based treatments, i.e., low drug solubility, poor biodistribution and low bioavailability, can be outlined through the integration into polymer/LDH nanocomposites. Therefore, it can potentialize the performance of the drug. Polymer/LDH composites for the ocular route assist local maintenance of active principles and can contribute to improving their permeation on the eye surface. Transdermal applications of LDH-based composites can be achieved by exploring diffusion processes from the external structure of the skin or by piercing the skin using an array of microneedles, for example.

LDH-based materials exhibit interesting features in different composite formulations because of their tunable composition and the variety of characteristics that their presence can produce in these composites, depending on their biomedical application. The polymer-LDH interactions also present a crucial role in the design of novel materials giving the initial properties of the organic phase, which can be retained or modified by the presence of the inorganic structure. Especially regarding the production of devices to assist wound healing and bone-related therapies, the choice of the polymer and the LDH composition is crucial for the direction of new tissues formation, and bone structure growth since metal ions from the LDH structure (i.e., Mg^2+^, Zn^2+^, Fe^3+^) have a role in the metabolism of cells involved in wound healing and COL neogenesis, and to possess osteogenic properties. All these characteristics indicate a feasible use of polymer/LDH composites with a tremendous potential breakthrough in the life quality of people suffering from severe diseases which can leave permanent side-effects or be lethal, such as diabetes and cancer, and also by the loss of function of organs and tissues resulting from accidents, diseases, and aging.

Although the combination of LDH and polymers clearly offers advantages in combining and complementing properties of each other, polymer/LDH composites still face challenges for both drug delivery and tissue engineering. In the case of oral administration, although the obstacle of sensitivity of LDH in acidic environments seems to be overcome with core-shell techniques using gastro-resistant polymers, the current challenge consists in overpassing the biological barriers, which can drastically decrease the bioavailability of the encapsulated drug due to changes in colloidal stability and/or enzymatic degradation of the LDH-based nanocomposite. However, recent advances have been widely explored to modulate drug release and create new smart and effective oral systems to increase bioavailability and decrease possible side effects, such as pH stimuli and magnetic or chemophotothermal therapies. On the other hand, intravenous administration routes are limited due to the difficulty in producing stable LDH suspension able to safely travel through the bloodstream and reach specific cells (systemic-targeted delivery). For tissue engineering, the main challenge resides in the dynamism of the wound environment or local damage, whose physical structure, chemical, and biological entities present chance according to the progression of the healing process and the multiple processes involved. Thus, polymer/LDH composites must be envisaged as smart multi-action materials engendered to follow each step of healing.

## Figures and Tables

**Table 1:** Review papers focusing on nanocomposites based on polymers and layered double hydroxides for pharmaceutical and medical applications published in 2017–2022.

Title	Year	Ref.
Nanoarchitectured two-dimensional layered double hydroxides-based nanocomposites for biomedical applications	2022	[167]
Layered double hydroxide applications in biomedical implants	2022	[168]
Surface modification of two-dimensional layered double hydroxide nanoparticles with biopolymers for biomedical application	2022	[159]
Topology dependent modification of layered double hydroxide for therapeutic and diagnostic platform	2022	[163]
Layered double hydroxide-based nanocomposite scaffolds in tissue engineering applications	2021	[169]
Advanced drug delivery applications of layered double hydroxide	2021	[170]
Recent advancements in synthesis and drug delivery utilization of polysaccharides-based nanocomposites: The important role of nanoparticles and layered double hydroxides	2021	[171]
2D Layered Double Hydroxide Nanoparticles: Recent Progress toward Preclinical/Clinical Nanomedicine	2019	[172]
Layered double hydroxide nanostructures and nanocomposites for biomedical applications	2019	[157]
Layered double hydroxide based bionanocomposites	2019	[173]
Layered double hydroxides: A brief review from fundamentals to application as evolving biomaterials	2018	[15]
Hybrid nanocomposites of layered double hydroxides: an update of their biological applications and future prospects	2017	[18]

## Data Availability

Not applicable.
